# Irrigated agriculture influences selenium levels in an endangered marsh bird

**DOI:** 10.1007/s10661-025-14533-1

**Published:** 2025-09-24

**Authors:** Cydney M. Yost, Kathryn M. Sliwa, Razia Shafique-Sabir, Jonathan Shore, Courtney J. Conway

**Affiliations:** 1 Idaho Cooperative Fish and Wildlife Research Unit, University of Idaho, Moscow, ID USA; 2 United States Fish and Wildlife Service, Sonny Bono Salton Sea National Wildlife Refuge, Calipatria, CA USA; 3 United States Geological Survey, Idaho Cooperative Fish and Wildlife Research Unit, University of Idaho, Moscow, ID USA

**Keywords:** Selenium bioaccumulation, Yuma Ridgway’s rail, Irrigated agriculture, Agricultural run-off, Salton Sea, Wetland contamination

## Abstract

Selenium bioaccumulation in aquatic food webs poses risks to wildlife, particularly in wetlands receiving irrigation runoff. The Salton Sea, California’s largest lake, is primarily sustained by agricultural drainage. This drainage creates wetland habitat along the lakeshore that many bird species depend on, including the federally endangered Yuma Ridgway’s rail (*Rallus obsoletus yumanensis*). However, these marshes may pose an ecological trap – attracting rails despite high selenium exposure. We captured rails during the 2020–2023 breeding seasons and compared rail selenium levels within three types of marshes (fed with irrigation runoff, Colorado River water, or groundwater). We collected blood, breast feathers, and head feathers of rails in all three water sources for selenium comparisons. We tagged adult rails with GPS transmitters to locate nests and foraging locations where we collected eggshells, unhatched eggs, and prey. We assessed selenium exposure by collecting multiple prey species commonly eaten by rails in all three water sources. Selenium concentrations varied among sampling locations. Selenium concentrations in most sample types were predominately influenced by water source and marsh inflow velocity (sometimes in combination with marsh size). Distance to inflow, however, did not influence selenium concentrations in any sample type. Selenium concentrations were highest in agricultural-fed marshes compared to river-fed and spring-fed marshes. Increased marsh inflow velocities resulted in lower selenium concentrations. Given the risk of an ecological trap, our results suggest that supplementing wetlands with Colorado River water could mitigate selenium bioaccumulation in Yuma Ridgway’s rails.

**Supplementary information:**

The online version contains supplementary material available at 10.1007/s10661-025-14533-1.

## Introduction

Irrigated agriculture can significantly alter the natural hydrologic regime within a watershed, affecting the timing, flow, and distribution of water, especially in arid and semiarid regions of the U.S. (McCarthy & Johnson, 2009). Drains that transport agricultural runoff are intended to maintain agricultural productivity, but they often result in minimal or no natural dilution, causing pollutants to flow directly into receiving water bodies (McCarthy & Johnson, 2009). The propensity for selenium to accumulate to abnormally high concentrations in agricultural drainage water, especially in arid regions, has become a growing concern in North America (Garone, 1999; Ohlendorf, 2002, 2011; Ohlendorf et al., 2020; Seiler et al., 2003). Selenium is a naturally occurring element found mainly in sedimentary rocks and soil deposits from the Late Cretaceous Epoch (100.5–66 mya). Late Cretaceous soil comprises approximately 17% of the western United States and is often irrigated for agricultural production in arid and semiarid regions (Popkin, 1986; Seiler et al., 2003). Low annual precipitation and high evaporation in arid climates likely intensify selenium bioaccumulation (Seiler et al., 2003), particularly during the spring and summer months when the demand for irrigation is highest, and wetland birds are concurrently breeding in North America.

Selenium can bioaccumulate in the aquatic food web of wetlands fed by agricultural runoff, and high selenium concentrations can reduce the fitness of wetland-dependent animals. For example, high selenium levels infamously caused lower egg hatchability, developmental abnormalities, and increased adult mortality in wetland birds at Kesterson Reservoir in the San Joaquin Valley of California (Garone, 1999; Ohlendorf, 2002, 2011; Ohlendorf et al., 2020). The large-scale mortality event at Kesterson in the 1980’s was followed by other well-documented cases of selenium-caused abnormalities and highlighted other potential wetland regions at risk of selenium toxicity, including wetlands throughout the Lower Colorado River Basin (Seiler et al., 2003).

The Lower Colorado River Basin includes substantial areas of former desert ecosystems that have been converted to irrigated agriculture. The Yuma Ridgway’s rail (*Rallus obsoletus yumanensis*) is a federally endangered bird endemic to wetlands throughout the Lower Colorado River Basin in the southwestern U.S. and northwestern Mexico (USFWS, 1967, 2010; Conway & Eddleman, 2000; Conway et al., 2010; Eddleman & Conway, 2020). These endangered rails depend on emergent wetlands that are separated by non-habitat (i.e., upland desert vegetation, agriculture, urban development) within their limited geographic distribution (Conway et al., 2010; Harrity et al., 2020). Irrigated agriculture has become common in the region (Norton et al., 2021), often immediately adjacent to the fragmented wetlands used by rails. Hence, many of the wetlands used by this endangered rail contain runoff from adjacent irrigated agricultural fields. Elevated selenium concentrations were reported in Yuma Ridgway’s rail tissues and their primary prey, including aquatic invertebrates and small fish (Eddleman, 1989; Ohmart & Tomlinson, 1977) at the Salton Sea in 1999, 2008–2009, and 2016 (King et al., 2000; McKernan et al., 2016; Ricca et al., 2022). However, annual shifts in water use may have influenced selenium exposure in recent years. Consequently, several state and federal agencies have created restored wetlands fed with Colorado River water (rather than runoff from irrigated agriculture) specifically to benefit Yuma Ridgway’s rail populations (Ricca et al., 2022).

Salton Sea wetlands, in southeastern California, support one of two core Yuma Ridgway’s rail populations in the U.S., and the amount of Colorado River water supplied to agricultural fields in the Imperial Valley has recently been reduced (Coachella Valley Water District et al., 2002), resulting in the shrinking of the Salton Sea and the emergence of newly exposed playa (Barnum et al., 2017). Yuma Ridgway’s rails use these newly established marshes in the open playa at agricultural drainage outlets (hereafter ag-fed marshes), expanding the habitat patches available to Yuma Ridgway’s rails in the region (Harrity et al., 2020). Management agencies are concerned that ag-fed marshes could act as an ecological trap, luring rails away from marshes fed with Colorado River (hereafter river-fed marshes) or spring water (hereafter spring-fed marshes), marshes that likely have lower selenium levels than ag-fed marshes (Bryon & Ohlendorf, 2007; USFWS, 2010; Ricca et al., 2022).

The objective of our study was to compare selenium concentrations in Yuma Ridgway’s rails among three types of marshes based on their water sources (i.e., ag-fed, river-fed, and spring-fed marshes). We tested four predictions: 1) Yuma Ridgway’s rails captured in ag-fed marshes would have higher selenium concentrations than Yuma Ridgway’s rails captured in river-fed and spring-fed marshes, 2) within each marsh, selenium concentrations would decline as inflow velocity increased, as faster moving water may reduce selenium accumulation by dispersing contaminants more evenly throughout each marsh, 3) selenium concentrations would decline as marsh size increased, as the element can dissipate over a larger area, reducing localized accumulation, and 4) in rail eggs and rail prey, selenium concentrations would decrease with increasing distance between a nest or prey sampling location and each marsh’s inflow location, as selenium may settle or be absorbed by vegetation and sediment closer to the inflow source, reducing accumulation of selenium further into the marsh.

## Materials and methods

### Study area

**Fig. 1:**
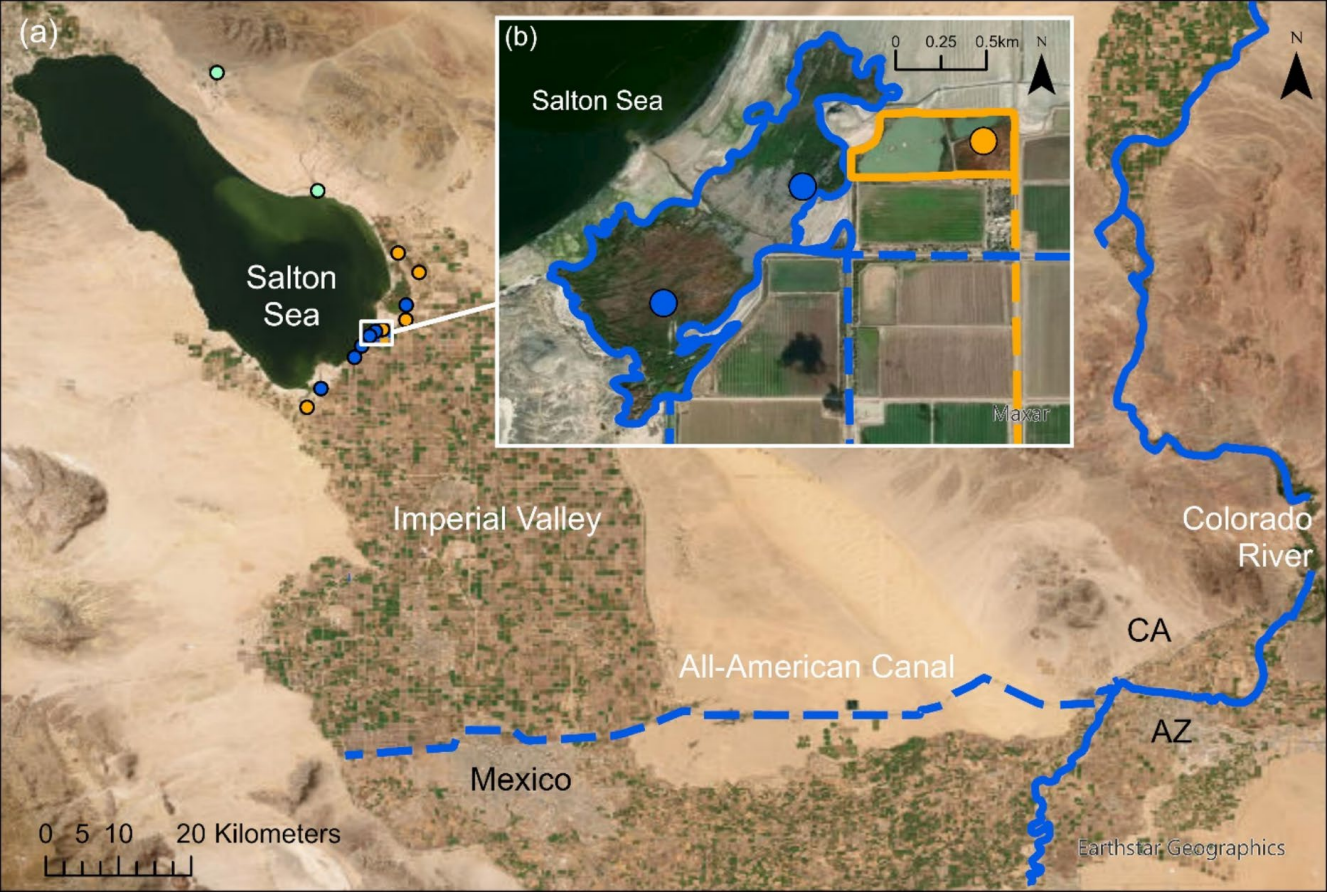
Map (a) depicts the All-American Canal (dashed blue line), which supplies Colorado River water (solid blue line) to the Imperial Valley from the south (ESRI, 2010). Map (a) also depicts marshes where Yuma Ridgway’s rails were captured around the Salton Sea (California, USA) during the 2020–2023 breeding seasons; green = spring-fed marsh, orange = river-fed marsh, and blue = ag-fed marsh. Map (b) shows a close-up of two adjacent marshes (solid lines) fed by agricultural drains (blue dashed lines) or Colorado River water (orange dashed line)

California provides 75% of the U.S. lettuce supply, with the Imperial Valley being the second largest producer in the state (California Department of Food and Agriculture, 2017). Additionally, the Imperial Valley produces 67% of the winter vegetables consumed in the U.S. (Imperial County Agricultural Commission, 2020). Within California, the Imperial Valley is the sole producer of sugar beets and accounts for 21.3% of alfalfa hay production for the state (Imperial County Agricultural Commission, 2020, 2021, 2022, 2023). The Imperial Valley’s agricultural production requires massive quantities of water. Colorado River water via the All-American Canal is the primary water source for roughly 200,000 ha of agricultural fields in the Imperial Valley (Imperial Irrigation District, 2024; Fig. 1). The All-American Canal is the largest irrigation canal in the U.S., transporting an average of 429 m^3^/s of water to irrigated agricultural fields in the Imperial Valley (Imperial Irrigation District, 2024). Irrigation in the valley relies on a gravity-flow system, with agricultural runoff draining directly into wetlands surrounding the Salton Sea. Ag-fed wetlands are the predominant wetland type in the region, primarily receiving agricultural runoff, but some also receive surplus Colorado River water not used for irrigation. The U.S. Fish and Wildlife Service (USFWS) and California Department of Fish and Wildlife (CDFW) purchase Colorado River water (i.e., unused irrigation water) to support river-fed marshes including marshes within Sonny Bono Salton Sea National Wildlife Refuge and Wister Wildlife Management Area. Several marshes in the region (at Bombay Beach and Dos Palmas Preserve) are maintained by spring water. All marshes were 64–69 m below sea level (Fig. 1). Maximum daily ambient temperatures in the Imperial Valley averaged between 25° C in March and 43° C in July across all four years of the study (2020–2023; NOAA, 2024). We calculated the area of each marsh (in hectares) using ArcGIS Pro (ESRI, 2024).

### Yuma Ridgway’s rail captures and tissue sample collection

We captured Yuma Ridgway’s rails from March–July for each of four years (2020–2023) in marshes adjacent to the Salton Sea, California (Fig. 1). We used annual Yuma Ridgway’s rail survey results to identify rail capturing locations (N. Engelmann, USFWS, unpublished data). We placed carpet traps (Harrity & Conway, 2020a) in areas where we heard Yuma Ridgway’s rails calling and broadcasted recorded Yuma Ridgway’s rail vocalizations (Conway, 2011) to attract birds to our traps. We determined the sex and age of each Yuma Ridgway’s rail that we captured based on morphometric measurements and plumage conditions (Eddleman & Conway, 2020; Harrity et al., 2021). We collected blood, breast feather (2020 and 2021), and head feather (2021 only) samples from a subset of rails we captured to document differences in selenium exposure among the three tissue types. Among our sample types, blood represents the most recent dietary uptake and the fastest turnover rate of selenium (Burger et al., 2018), whereas feathers represent selenium in the diet during the temporal window of feather growth (i.e., a two- or three-week window when the sampled feather last molted or regrew; Ackerman et al., 2016; Burger & Gochfeld, 1997). We drew ≤ 0.5 ml of blood from the metatarsal vein of Yuma Ridgway’s rails with a 26-gauge needle and 1-ml syringe. We stored ≤ 0.07 ml of blood in 1-ml Microtainer tubes containing Queen’s Lysis Buffer to verify sex via DNA markers and stored the remaining blood in 1-ml EDTA Microtainer tubes for selenium concentration analysis. We extracted DNA with a DNeasy Tissue Kit (Qiagen Inc., Valencia, CA, USA). We followed the methods outlined by Griffiths et al. (1998), with adjustments implemented by Harrity et al. (2021), to enhance the DNA amplification process for the sex identification of Yuma Ridgway’s rails. We plucked three breast feathers (≤ 20 mg/feather) and three head feathers (≤ 2 mg/feather) for selenium concentration analysis. We stored blood in buffer samples at room temperature and all other tissue samples in a freezer at −21° C.

From 2020 to 2023, we attached solar-powered PinPoint transmitters (Lotek Wireless Inc., Newmarket, ON, Canada) to Yuma Ridgway’s rails that were large enough to ensure that the combined weight of the transmitter and harness equipment was ≤ 5% of each bird’s total mass. We used transmitters to locate rail nesting and foraging locations. The transmitters weighed 6.0 to 6.5 g and were equipped with small solar panels for recharging. We used Spectra-ribbon (Bally Ribbon Mills, Bally, PA, USA; Dwyer, 1972) for harness material to attach the PinPoint transmitters to rails. After recording every third successful GPS location, the transmitters uploaded location data to an online server via the Argos satellite network. We programmed transmitters to record 2–3 GPS locations per day during the breeding season. During the non-breeding season, we adjusted the schedule to capture one GPS location every other day. Since the transmitters relied on solar power and rails occupy areas with dense vegetation, this approach balanced data collection while conserving battery life across seasons.

### Yuma Ridgway’s rail nests

We used GPS data from satellite transmitters attached to adult Yuma Ridgway’s rails to monitor nesting activity remotely during all four years of the study. We strived to visit nests only after each nesting attempt was complete to minimize disturbance to active nests. We only approached a suspected nesting location if: 1) GPS locations indicated the adult had spent ≥ 3 consecutive days away from the nest, or 2) more than 28 days had passed since estimated nest initiation (incubation typically lasts 23–28 days; Eddleman, 1989).

We used a GPS receiver to record nest coordinates once we determined that a nest was no longer active, and any remaining unhatched eggs were nonviable. From 2020 to 2022, we collected unhatched eggs and shell fragments from within or around the immediate nesting locations. Rallidae species often use their bills to reposition eggs (Kaufmann, 1989) and may remove eggshells after hatching presumably to reduce the probability of attracting predators. Eggshell fragments from hatched or depredated eggs often settle into the layers of cattail that form the nest or fall below the nest bowl. Post-hatch eggshells have been used in a number of biomonitoring studies, providing insights into contaminant exposure in avian species (Kitowski et al., 2018). Given their potential ecological significance, we made a concerted effort to recover all remaining eggshell fragments (*n* = 50, mean = 880 mg, range = 50–4080 mg) from rail nests. We rinsed unhatched eggs and eggshell fragments of hatched or depredated eggs in deionized water. Most of the eggshell fragments were very small (*n* = 39, mean = 4.95 mm, range = 1–10 mm). Hence, we were unable to determine whether small bits of the eggshell membranes were still attached to each eggshell fragment. We transferred egg contents (separate from their eggshells and outer eggshell membranes) to individual, sterilized jars. If a nest had hatched eggs plus one unhatched egg, three samples from an individual nest were submitted for selenium analysis. We air-dried all eggshell samples and then weighed and stored them in new, sterilized jars. We stored eggshell samples and egg content samples at −21° C.

### Yuma Ridgway’s rail prey

Aquatic invertebrates and small fish are the primary prey of Yuma Ridgway’s rails (Eddleman, 1989; Ohmart & Tomlinson, 1977). We collected western mosquitofish (*Gambusia affinis*) and red swamp crayfish (*Procambarus clarkii*) during three breeding seasons (2020–2022) to compare selenium concentrations in Yuma Ridgway’s rail prey items among the three types of marshes (fed by the three different water sources). We expanded minnow trapping in 2022 and 2023 to include other potential prey including sailfin molly (*Poecilia latipinna*), common carp (*Cyprinus carpio*), bluegill sunfish (*Lepomis macrochirus*), Mozambique tilapia (*Oreochromis mossambicus*), red shiner (*Cyprinella lutrensis*), largemouth bass (*Micropterus salmoides*), American bullfrog tadpoles (*Lithobates catesbeianus*), shrimp (*Palaemonidae* sp.), and other arthropods (order Coleoptera, family Belostomatidae, family Corixidae) to better capture the potential diet and selenium intake of Yuma Ridgway’s rails. We collected a subset of all species captured in 2022 for selenium analysis in addition to prey from 2020 and 2021. The number of each species we trapped in 2022 and 2023 were used to estimate and compare prey abundance among spring-fed, river-fed, and ag-fed marshes.

We captured prey throughout each marsh to determine how selenium concentration was associated with distance to inflow. We sampled prey at known water inflows and outflows of each marsh. We randomly chose 1–5 GPS points within each GPS-tagged rails’ home range and randomly selected 1–5 GPS points outside of any rail home range to sample prey. We trapped all prey by sinking 1–3 minnow traps at designated locations (2020–2023). To attract Yuma Ridgway’s rail prey, we placed deli meat in perforated plastic water bottles within the minnow traps. The perforated bottles allowed the scent of the deli meat to enter the water while preventing the prey from consuming the bait, reducing the likelihood that the sampled prey ingested bait, artificially changing selenium levels in Yuma Ridgway’s rail prey samples. We checked the minnow traps every four hours to minimize mortality risk to non-target species. Mosquitofish and crayfish varied in size. To ensure we had a large enough sample for selenium analysis, we stopped sampling once we captured at least six mosquitofish and 1–5 crayfish at each sampling point. When traps consisted of more than six fish or more than five crayfish, we made sure to collect prey of variable sizes for the most accurate average selenium value. We used sweep nets to catch mosquitofish if minnow traps were ineffective because mosquitofish were the only fish prey that we sought during the first two years of the study. We stored all prey samples collected at −21° C. The selenium levels in prey collected from 2020–2022 represent the average concentrations of all individual species collected from a specific sampling point.

### Selenium analysis

We sent Yuma Ridgway’s rail tissue, egg, and prey samples collected from marshes of all three water sources to the Trace Element Research Lab at Texas A&M University for selenium analysis. The lab followed methods under the National Oceanic and Atmospheric Administration (NOAA; Kimbrough & Lauenstein, 2006) for sample preparation and processing, which included cleaning with deionized water to remove environmental particles (feathers only) and acid digestion to decompose organic material and release selenium. Samples were then freeze-dried to remove moisture while preserving the integrity of selenium ions. Homogenization ensured a consistent mixture for accurate selenium concentration measurements. The Trace Element Research Lab used inductively coupled plasma mass spectroscopy with a Perkin Elmer NexION 2000 C to measure selenium concentrations, employing ionization and mass spectrometry for precise detection of trace elements. All results presented reflect dry weight measurements in parts per million (ppm = µg/g; Ohlendorf, 2003). We measured selenium concentrations in the same units (ppm) among all sample types so we could directly compare how different environmental factors affected selenium concentrations among sample types.

### Data analysis

We used selenium concentrations in blood, breast feathers, and head feathers of Yuma Ridgway’s rails to address our first three predictions on water source, inflow velocity, and marsh size. We used the function ‘lmer’ from the package ‘lme4’ (Bates et al., 2015, Version 1.1–35.5) in the R statistical program (R Core Team, 2024, Version 4.4.2) to build blood and breast feather selenium models in a mixed-effects framework with year as the random effect. We used the ‘lm’ function from base R (R Core Team, 2024, Version 4.4.2) to model log-transformed selenium concentrations in head feathers because we only had one year of data for head feathers. We used a two-phase model-selection approach to examine factors influencing variation in selenium concentrations in Yuma Ridgway’s rail tissues. In phase 1, we selected sex, age, and mass as explanatory variables to account for potential variation in dietary behavior. We also included two-way interactions (sex*mass, age*mass), excluding sex*age due to the limited sample size of juveniles. We incorporated these variables into a set of eight candidate models for each tissue type. We used Akaike’s Information Criterion adjusted for a small sample size (AICc; Burnham & Anderson, 2002) to rank the candidate models and used the top model from phase 1 (for each tissue type) as the informed null model for phase 2 of model selection. In phase 2, we added water characteristics (water source, with ag-fed as the reference category; marsh inflow velocity), marsh characteristics (marsh size), and a two-way interaction (marsh inflow velocity*marsh size) as potential explanatory variables and examined a suite of eight candidate models for each tissue type. Estimated inflow velocities for river-fed and ag-fed marshes were provided by the Imperial Irrigation District (Olivia Alcaraz, IID, unpublished data); however, no inflow velocity data were available for spring-fed marshes. We used a two-week rolling average of inflow velocity (m^3^/s) associated with rail capture date. We used Akaike’s Information Criterion adjusted for a small sample size (AICc; Burnham & Anderson, 2002) to rank the candidate models and considered models with an ΔAICc value ≤ 2 as a top model.

We used selenium concentrations in rail eggs and prey to address all four of our predictions (i.e., water source, inflow velocity, marsh size, distance to inflow). We used the function ‘lmer’ from the package ‘lme4’ (Bates et al., 2015, Version 1.1–35.5) in the R statistical program (R Core Team, 2024, Version 4.4.2) to build egg and prey selenium models in a mixed-effects framework with year as the random effect. To examine the factors that influence variation in selenium concentrations in Yuma Ridgway’s rail eggshells, egg contents, and prey, we used a one-phase model selection approach including four explanatory variables (water source, with ag-fed as the reference category; marsh inflow velocity, marsh size, and distance of nest or prey sampling location to the marsh inflow), and a two-way interaction (marsh inflow velocity*marsh size). We used a one-phase approach for this analysis to directly assess the effects of environmental variables. We used a two-week rolling average of inflow velocity associated with prey collection date or nest end date (i.e., the last day a GPS-tagged rail returned to the nest during the nesting attempt). We examined a suite of 16 candidate models for each sample type and used partial-effects plots to examine the relationships between selenium concentration and our explanatory variables included in the top model. We used Akaike’s Information Criterion adjusted for a small sample size (AICc; Burnham & Anderson, 2002) to rank the candidate models and considered models with an ΔAICc value ≤ 2 as a top model.

## Results

### Yuma Ridgway’s rail captures

We captured 203 Yuma Ridgway’s rails (173 adults, 30 juveniles) across 13 marshes (2 spring-fed, 5 river-fed, 6 ag-fed; 2020–2023). From 2020 to 2021, we collected blood from 118 Yuma Ridgway’s rails, breast feathers from 119 rails, and head feathers (2021 only) from 77 rails for selenium analysis (Table 1; Online Resource 1-Table 4). From 2020 to 2023, we deployed GPS transmitters on 92 adult rails (39 females, 53 males) to locate nesting and foraging sites.

**Table 1:** We collected selenium concentration data from Yuma Ridgway’s rail blood, breast feathers, head feathers, egg contents, eggshells, and prey samples within marshes of three different water sources (spring-fed, river-fed, and ag-fed) at the Salton Sea, California, USA. We report the number of samples collected (*n*), geometric mean (GM), geometric standard deviation (GSD), and range of selenium concentrations in each sample type (ppm dry weight). Prey samples submitted for selenium analysis from all three breeding seasons include western mosquitofish (*Gambusia affinis*) and red swamp crayfish (*Procambarus clarkii*). Additional prey samples submitted for selenium analysis in 2022 include sailfin molly (*Poecilia latipinna*), common carp (*Cyprinus carpio*), bluegill sunfish (*Lepomis macrochirus*), Mozambique tilapia (*Oreochromis mossambicus*), and several other common fish (*Cyprinella*, *Micropterus*, *Morone* spp), shrimp (*Palaemonidae* sp), American bullfrog tadpoles (*Lithobates catesbeianus*), and several common insect and beetle taxa (Belostomatidae, Corixidae spp, *Coleoptera* sp)

	Spring-fed	River-fed	Ag-fed	Total
	GM ± GSD (Range, *n*)	GM ± GSD (Range, *n*)	GM ± GSD (Range, *n*)	GM ± GSD (Range, *n*)
**Rail Tissues**				
*blood*^*a*^	6.44 ± 1.59 (5.01–18.00, 7)	7.85 ± 1.70 (1.02–27.80, 52)	11.60 ± 1.46 (5.01–27.20, 59)	9.43 ± 1.65 (1.02–27.80, 118)
adults	6.66 ± 1.65 (5.01–18.00, 6)	8.17 ± 1.61 (2.30–27.80, 46)	11.57 ± 1.48 (5.01–27.20, 49)	9.56 ± 1.61 (2.30–27.80, 101)
juveniles	5.30 (1)	5.76 ± 2.35 (1.02–9.29, 6)	11.73 ± 1.33 (7.83–18.60, 10)	8.71 ± 1.89 (1.02–18.60, 17)
*breast feathers*^*a*^	3.41 ± 2.17 (1.37–8.47, 6)	4.57 ± 1.83 (1.10–14.60, 51)	5.90 ± 1.81 (1.23–15.30, 62)	5.15 ± 1.86 (1.10–15.30, 119)
adults	3.41 ± 2.17 (1.37–8.47, 6)	4.23 ± 1.85 (1.10–14.60, 46)	5.63 ± 1.83 (1.23–15.00, 52)	4.92 ± 1.88 (1.10–15.00, 104)
juveniles	-	6.19 ± 1.55 (3.64–9.85, 5)	7.59 ± 1.60 (3.77–15.30, 10)	7.09 ± 1.58 (3.64–15.30, 15)
*head feathers*^*b*^	3.99 ± 3.22 (1.84–38.00, 6)	7.93 ± 1.93 (3.18–27.00, 31)	11.51 ± 1.93 (2.50–42.00, 40)	9.12 ± 2.13 (1.84–42.00, 77)
adults	3.99 ± 3.22 (1.84–38.00, 6)	7.93 ± 1.93 (3.18–27.00, 31)	11.06 ± 1.96 (2.50–42.00, 36)	8.83 ± 2.14 (1.84–42.00, 73)
juveniles	-	-	16.51 ± 1.56 (8.92–24.70, 4)	16.51 ± 1.56 (8.92–24.70, 4)
**Rail Eggs**				
*eggshells*^*c*^	0.51 ± 1.88 (0.25–0.78, 3)	0.49 ± 1.90 (0.17–2.67, 20)	0.65 ± 2.03 (0.22–2.76, 27)	0.57 ± 1.97 (0.17–2.76, 50)
*egg contents*^*d*^	-	2.54 ± 1.78 (1.69–9.10, 7)	5.10 ± 1.51 (2.84–8.34, 8)	3.69 ± 1.82 (1.69–9.10, 15)
**Prey**				
*mosquitofish*^*c*^	3.66 ± 2.32 (0.47–11.20, 27)	3.78 ± 1.86 (0.52–15.60, 97)	6.24 ± 1.57 (1.84–16.30, 93)	4.67 ± 1.89 (0.47–16.30, 217)
*crayfish*^*c*^	3.47 ± 1.44 (2.15–6.07, 16)	2.62 ± 1.79 (0.64–12.50, 157)	4.16 ± 1.39 (1.43–10.10, 76)	3.07 ± 1.73 (0.64–12.50, 249)
*sailfin molly*^*e*^	3.97 ± 1.59 (2.13–6.57, 4)	3.97 ± 1.46 (1.99–5.69, 7)	9.52 ± 2.16 (2.05–19.40, 10)	6.02 ± 2.09 (1.99–19.40, 21)
*carp*^*e*^	-	4.16 ± 3.04 (0.13–8.80, 12)	-	4.16 ± 3.04 (0.13–8.80, 12)
*bluegill*^*e*^	-	2.82 ± 1.24 (2.16–3.51, 6)	-	2.82 ± 1.24 (2.16–3.51, 6)
*tilapia*^*e*^	-	-	10.02 ± 1.26 (7.79–13.70, 4)	10.02 ± 1.26 (7.79–13.70, 4)
*other fish*^*e*^	-	5.87 (1)	4.89 ± 1.37 (3.92–6.11, 2)	5.20 ± 1.28 (3.92–6.11, 3)
*shrimp*^*e*^	8.04 ± 1.15 (7.30–8.86, 2)	4.99 ± 1.65 (2.85–10.10, 6)	6.74 ± 1.45 (4.13–11.30, 14)	6.31 ± 1.51 (2.85–11.30, 22)
*tadpoles*^*e*^	10.77 ± 2.07 (4.81–19.80, 3)	5.46 ± 1.70 (1.81–11.70, 10)	7.87 ± 1.42 (5.83–11.60, 3)	6.64 ± 1.78 (1.81–19.80, 16)
*insects*^*e*^	2.78 ± 2.23 (1.58–4.90, 2)	2.82 ± 1.55 (1.82–5.37, 7)	3.41 ± 2.13 (1.22–11.80, 7)	3.06 ± 1.82 (1.22–11.80, 16)

Years sampled: ^a^2020–2021, ^b^2021, ^c^2020–2022, ^d^2021–2022, ^e^2022

### Yuma Ridgway’s rail nests

We found 77 Yuma Ridgway’s rail nests across 11 marshes (2 spring-fed, 5 river-fed, 4 ag-fed; 2020–2023), including 64 from GPS-tagged rails and 13 (17%) nests that we found incidentally (i.e., nests found without using GPS points from a GPS-tagged rail and unassigned to a specific banded bird). From 2020 to 2022, we collected eggshell or egg content samples from 38 nests for selenium analysis (50 eggshell samples; 10 whole eggs and 5 cracked eggs from which we collected eggshells and egg content; Table 1; Online Resource 2-Table 3). We collected eggshells from 28 nests where no unhatched eggs were detected, egg content from three nests where no other shells were present, and both egg content (from whole or cracked eggs) and eggshells from seven nests.

### Yuma Ridgway’s rail prey

We collected rail prey from 2020 to 2023. Between 2020 and 2022, we collected a subset of prey from 279 sampling locations (i.e., individual sets of minnow traps) within 12 marshes to submit for selenium analysis (2 spring-fed, 5 river-fed, 5 ag-fed; Table 1; Online Resource 3-Table 4). Of the 279 prey sampling locations for selenium analysis, 47% were within GPS-tagged Yuma Ridgway’s rail home ranges, 25% were at random points outside home ranges, 19% were at inflow locations (irrigation or river water), and 9% were at outflow canals. Across 2020 to 2022, most prey samples submitted for selenium analysis were invertebrates (crayfish: 45%, shrimp: 4%, arthropods: 3%) followed by fish (mosquitofish: 37%, other fish: 8%), and tadpoles (3%). In 2022 and 2023, we expanded the prey species we collected to estimate and compare species abundance among the three different water sources (Online Resource 3-Table 1). Among the three water sources, crayfish, sailfin molly, and shrimp made up a considerable proportion of the prey captured in ag-fed marshes (Online Resource 3-Table 1). Crayfish and shrimp also made up a considerable proportion of prey captured in river-fed marshes; however, mosquitofish made up the largest proportion of the prey composition in marshes of all three water sources (Online Resource 3-Table 1).

### Selenium analysis and modeling results

**Fig. 2:**
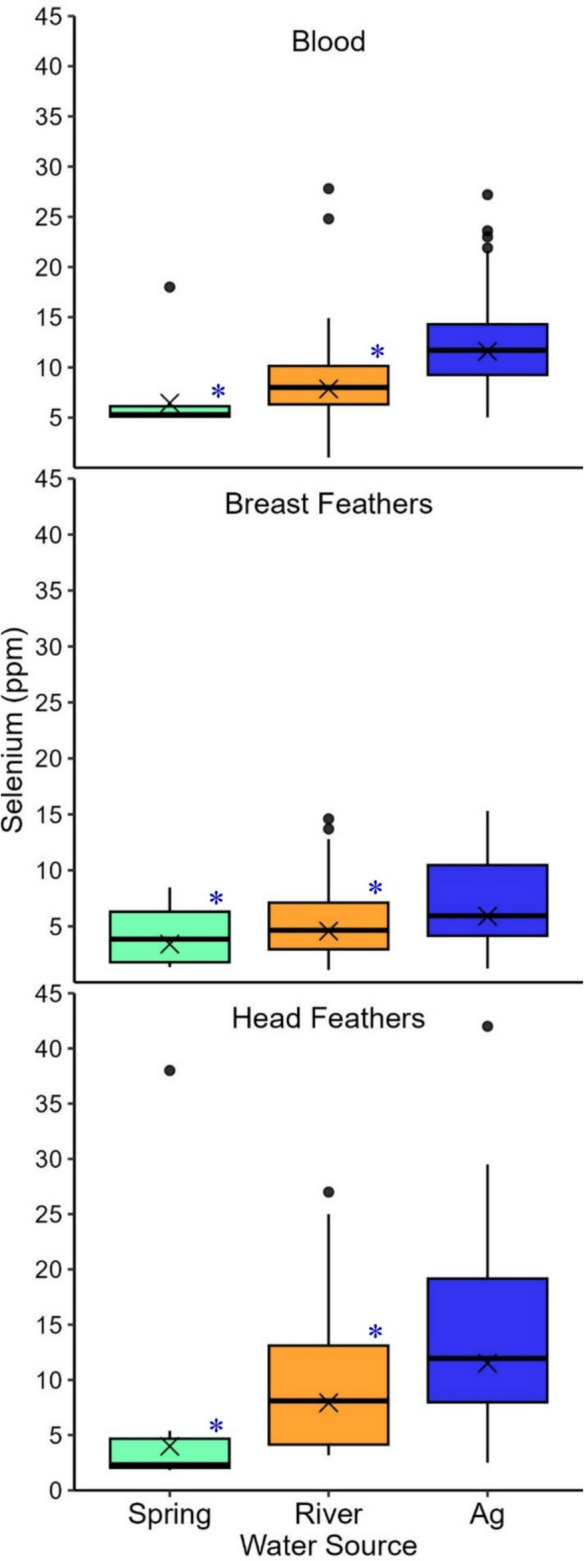
Selenium concentrations (ppm dw) in Yuma Ridgway’s rail (all ages combined) blood, breast feathers, and head feathers (2021 only) sampled from spring-fed (green), river-fed (orange), and ag-fed (blue) marshes at the Salton Sea, California, USA (2020–2021). Boxplots illustrate the 25th and 75th percentiles, with medians represented by lines inside each box. Whiskers extend to the 5th and 95th percentiles. Dots denote outliers and X’s indicate geometric means. Blue asterisks denote statistically significant differences between spring-fed or river-fed marshes and the reference category, ag-fed marshes

**Fig. 3:**
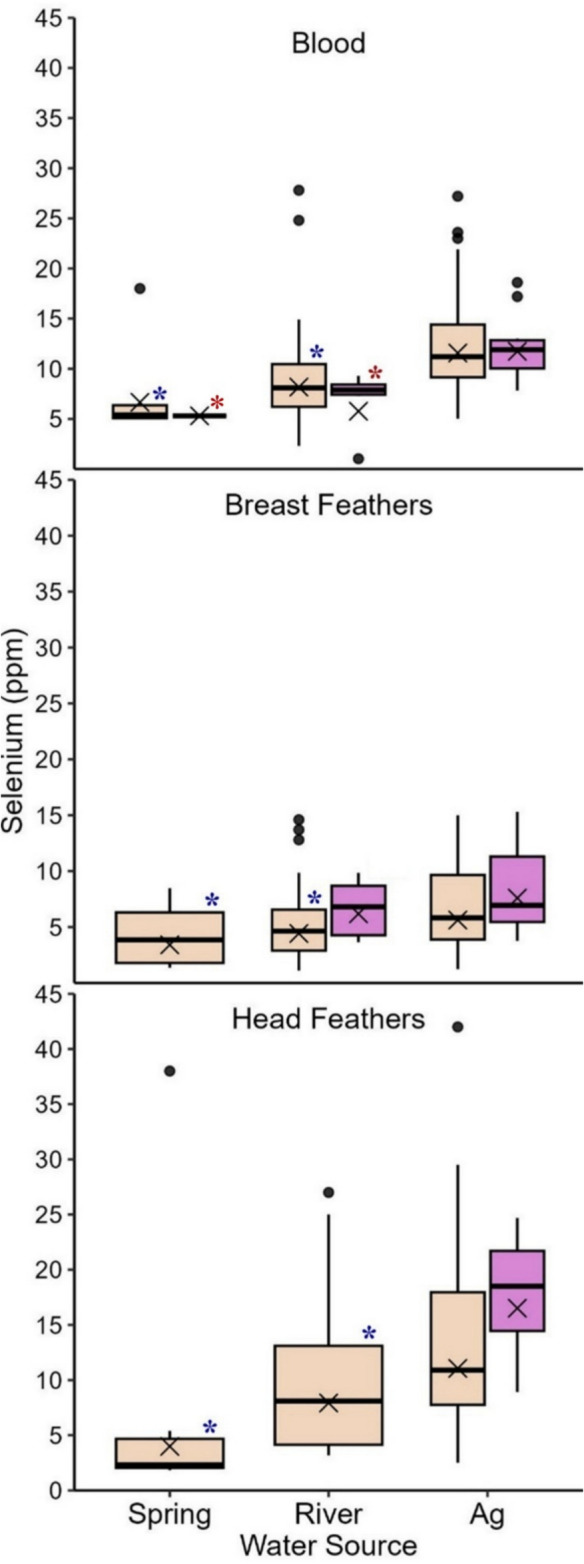
Selenium concentrations (ppm dw) in adult (beige) and juvenile (purple) Yuma Ridgway’s rail blood, breast feathers, and head feathers (2021 only) sampled from spring-fed, river-fed, and ag-fed marshes at the Salton Sea, California, USA (2020–2021). Boxplots illustrate the 25th and 75th percentiles, with medians represented by lines inside each box. Whiskers extend to the 5th and 95th percentiles. Dots denote outliers and X’s indicate geometric means. Blue (adult) and red (juvenile) asterisks denote statistically significant differences between spring-fed or river-fed marshes and the reference category, ag-fed marshes

**Fig. 4:**
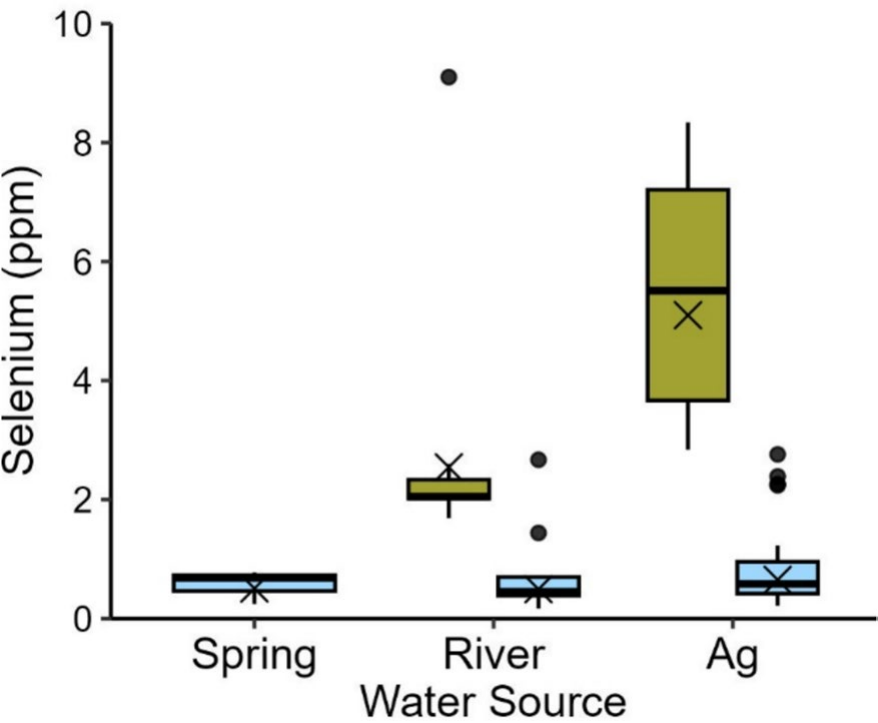
Selenium concentrations (ppm dw) in egg contents (gold) and eggshells (blue) sampled from Yuma Ridgway’s rail nests in spring-fed (shells only), river-fed, and ag-fed marshes at the Salton Sea, California, USA (2020–2022). Boxplots illustrate the 25th and 75th percentiles, with medians represented by lines inside each box. Whiskers extend to the 5th and 95th percentiles. Dots denote outliers and X’s indicate geometric means. No statistically significant differences were detected among eggshells or between egg contents of the differing water sources

**Fig. 5:**
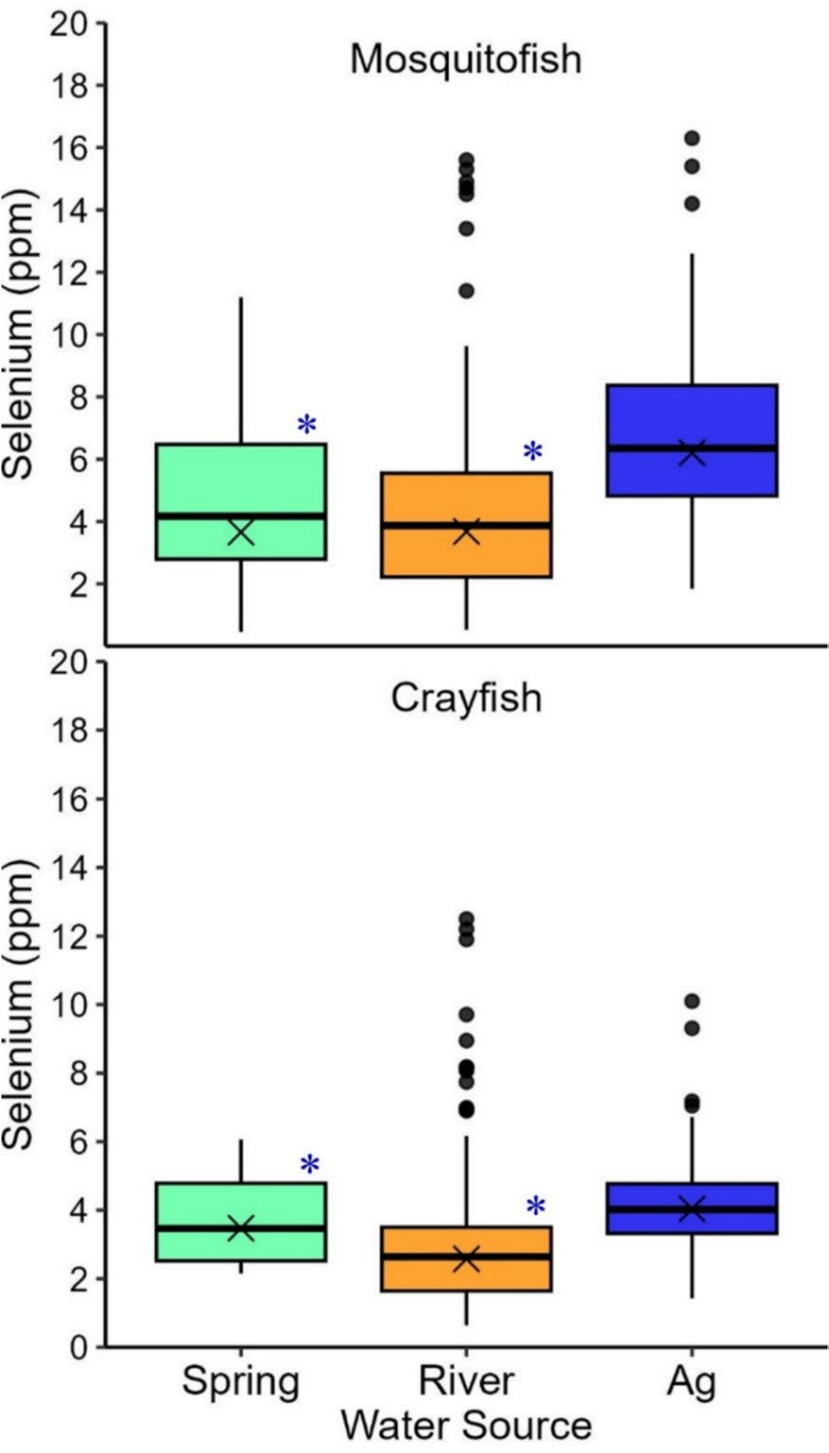
Selenium concentrations (ppm dw) in mosquitofish and crayfish sampled from spring-fed (green), river-fed (orange), and ag-fed (blue) marshes at the Salton Sea, California, USA (2020–2022). Boxplots illustrate the 25th and 75th percentiles, with medians represented by lines inside each box. Whiskers extend to the 5th and 95th percentiles. Dots denote outliers and X’s indicate geometric means. Blue asterisks denote statistically significant differences between spring-fed or river-fed marshes and the reference category, ag-fed marshes

Selenium concentrations differed among the rail tissue types and differed among the three water sources within tissue types (Tables 1 and 2; Fig. 2; Online Resource 1-Table 1a–3b). We also found some evidence that selenium levels of juvenile rails were higher than those of adult rails in the two feather types but not in blood samples (Tables 1 and 2; Fig. 3; Online Resource 1-Table 1b, 2b). Selenium concentrations differed in egg content but not eggshells among different water sources (Tables 1 and 2; Fig. 4; Online Resource 2-Table 1a, 2a). Selenium concentrations also differed among prey species and differed among the three water sources within prey species (Tables 1 and 2; Fig. 5; Online Resource 3-Table 2a–3b, Fig. 2).

Water source (spring-fed, river-fed, or ag-fed) and marsh inflow velocity explained variation in selenium concentrations for blood and feathers of Yuma Ridgway’s rails, but the relationships were stronger for blood (w_i_ = 0.97) than for feathers (Table 2; Online Resource 1-Table 1a, 2a, 3a). Between feather types, water source showed consistent support (breast feathers: w_i_ = 0.93; head feathers: w_i_ = 0.76), but inflow velocity was only moderately supported for breast feathers (w_i_ = 0.42; Table 2; Online Resource 1-Table 2a, 3a). Based on model outputs, selenium concentrations in blood, breast feathers, and head feathers of Yuma Ridgway’s rails were significantly higher in ag-fed marshes than spring-fed and river-fed marshes (Online Resource 1-Table 1b, 2b, 3b). Marsh inflow velocity was negatively associated with selenium concentrations in blood, but the relationship was not as strong in breast feathers (Table 2; Online Resource 1-Table 1a–2b). Selenium concentrations in blood varied between 1.02 ppm and 27.80 ppm at the lowest inflow velocities (≤ 0.1 m^2^/s) and between 9.03 ppm and 18 ppm at the highest inflow velocities (≥ 0.3 m^2^/s; Online Resource 1-Table 4, Fig. 1). Selenium concentrations in breast feathers varied between 1.10 ppm and 15.30 ppm at the lowest inflow velocities (≤ 0.1 m^2^/s) and between 4.85 ppm and 15 ppm at the highest inflow velocities (≥ 0.3 m^2^/s; Online Resource 1-Table 4, Fig. 1). Marsh size showed a weak negative association with breast and head feathers (Table 2; Online Resource 1-Table 2a, 3a, 3b). Selenium concentrations in breast feathers varied between 1.59 ppm and 15.30 ppm in the smallest marshes (≤ 12 ha) and between 1.72 ppm and 14.80 ppm in the largest marshes (≥ 856 ha; Online Resource 1-Table 4). Selenium concentrations in head feathers varied between 3.40 ppm and 25 ppm in the smallest marshes (≤ 12 ha) and between 2.50 ppm and 42 ppm in the largest marshes (≥ 856 ha; Online Resource 1-Table 4).

**Table 2:** Top models selected from separate Akaike’s Information Criterion comparisons adjusted for small sample sizes (AICc; Burnham & Anderson, 2002) to explain variation in selenium concentrations of Yuma Ridgway’s rail blood, breast feathers, head feathers, eggshells, egg content, and prey collected from the Salton Sea, California, USA. Explanatory variables considered include sex, age (juvenile vs. adult), water source (spring-fed, river-fed, or ag-fed), inflow velocity (two-week rolling average at the time of rail capture/sample collection), marsh size (total hectares of continuous cattail marsh), and distance to inflow (not applicable for rail tissue samples). ΔAICc = the difference in AICc values from the best-fitting model; w_i_ = Akaike weight of each model

	Top Models	ΔAIC_C_	w_i_
**Rail Tissues**			
*blood*^*a*^	sex + age + water source + velocity + (1|year)	0.00	0.97
*breast feathers*^*a*^	age + water source + velocity + (1|year)	0.00	0.42
	age + water source + marsh size + (1|year)	0.49	0.33
	age + water source + (1|year)	1.69	0.18
*head feathers*^*b*^	water source + marsh size	0.00	0.76
**Rail Eggs**			
*eggshells*^*c*^	null	0.00	0.52
	velocity + (1|year)	0.48	0.41
*egg contents*^*d*^	velocity + (1|year)	0.00	0.50
	water source + velocity + (1|year)	1.68	0.21
**Prey**^**c**^			
*mosquitofish*	water source + velocity + (1|year)	0.00	0.70
	water source + velocity + velocity*marsh size + (1|year)	1.78	0.29
*crayfish*	water source + velocity + (1|year)	0.00	0.94

Years sampled: ^a^2020–2021, ^b^2021, ^c^2020–2022, ^d^2021–2022

Water source (w_i_ = 0.21) and marsh inflow velocity (w_i_ = 0.71) explained variation in selenium concentrations for egg contents of Yuma Ridgway’s rails; however, only velocity helped explain variation in selenium concentrations of eggshells (w_i_ = 0.41; Table 2; Online Resource 2-Table 1a, 2a). Egg contents had higher average selenium concentrations than eggshell samples in river-fed and ag-fed marshes (Table 1; Fig. 4). We were unable to collect any egg contents from spring-fed marshes. Egg content selenium concentrations were not significantly different between river-fed and ag-fed marshes. However, the highest egg content (9.10 ppm) was collected from a nest in a river-fed marsh; and all other samples from river-fed marshes had lower selenium concentrations than those collected from ag-fed marshes (Table 1; Fig. 4). Selenium concentrations in eggshells did not differ among the three water sources, and selenium concentrations in eggshells and egg content were not associated with marsh size or distance to inflow (Table 2; Online Resource 2-Table 1a, 2a). However, egg content did exhibit a weak negative relationship with marsh inflow velocity (Table 2; Online Resource 2-Table 1a, 1b). Selenium concentrations in egg contents varied between 1.69 ppm and 9.10 ppm at the lowest inflow velocities (≤ 0.1 m^2^/s) and between 3.09 ppm and 3.86 ppm at the highest inflow velocities (≥ 0.2 m^2^/s; Online Resource 2-Table 3).

Selenium concentrations in mosquitofish and crayfish differed by water source and inflow velocity (mosquitofish w_i_ = 0.99; crayfish w_i_ = 0.94; Tables 1 and 2; Fig. 5; Online Resource 3-Table 2a–3b). Based on model outputs, mosquitofish and crayfish collected from ag-fed marshes had significantly higher selenium values than those collected from spring-fed and river-fed marshes (Online Resource 3-Table 2a–3b). Selenium concentrations in mosquitofish and crayfish were not associated with distance to inflow (Table 2; Online Resource 3-Table 2a, 3a). Selenium concentrations in both mosquitofish and crayfish were negatively correlated with inflow rate (Table 2; Online Resource 3-Table 2b, 3b). Selenium concentrations in mosquitofish varied between 0.47 ppm and 16.30 ppm at the lowest inflow velocities (≤ 0.1 m^2^/s) and between 1.84 ppm and 12.20 ppm at the highest inflow velocities (≥ 0.2 m^2^/s; Online Resource 3-Table 4, Fig. 1). Selenium concentrations in crayfish varied between 0.64 ppm and 12.50 ppm at the lowest inflow velocities (≤ 0.1 m^2^/s) and between 1.43 ppm and 7.18 ppm at the highest inflow velocities (≥ 0.2 m^2^/s; Online Resource 3-Table 4, Fig. 1). Mosquitofish relationship with inflow velocity varied inconsistently across different marsh sizes (Table 2; Online Resource 3-Table 2a). Selenium concentrations in mosquitofish varied between 1.35 ppm and 15.60 ppm in the smallest marshes (≤ 12 ha) and between 1.84 ppm and 16.30 ppm in the largest marshes (≥ 856 ha; Online Resource 3-Table 4). Among all prey collected in 2022, sailfin molly and Mozambique tilapia had the highest selenium concentrations of fish, and shrimp and tadpoles had the highest concentrations of non-fish sampled (Online Resource 3-Fig. 2).

All selenium values for rail tissues, eggs, and prey may be found in supplementary information (Online Resources 1, 2, and 3).

## Discussion

The Salton Sea provides habitat for hundreds of waterbird species and the area is a destination for recreational birders due to the diversity and abundance of birds that use the open water and wetlands of the area (USFWS, 2024). The Salton Sea and adjacent wetlands are fed by multiple water sources (i.e., agricultural runoff, Colorado River, and natural springs). Prior to our study, selenium concentrations in irrigated water at the Salton Sea were documented to increase 8- to 14-fold from concentrations in the Colorado River (Setmire & Schroeder, 1998). The bioaccumulation of selenium in ag-fed marshes poses a dietary exposure risk to wetland-obligate birds like Yuma Ridgway’s rails (Bruehler & de Peyster, 1999). Our study provides valuable insights into how selenium levels vary among Salton Sea marshes that receive agricultural drainage water compared to those fed directly by Colorado River water or natural spring water. Additionally, we shed light on the influence of water dynamics on selenium accumulation in the environment.

As we predicted, average selenium concentrations in Yuma Ridgway’s rail tissues, egg content, and prey collected from ag-fed marshes were higher than those collected in river-fed and spring-fed marshes. Our results corroborate a prior study on Yuma Ridgway’s rails at the Salton Sea that found similar patterns in rail tissues and prey between ag-fed and river-fed marshes in 2016 (Ricca et al., 2022). We built upon the previous study (Ricca et al., 2022) by capturing and GPS-tagging more rails, significantly increasing our blood and feather sample sizes. Additionally, we collected a wider variety of prey types. We also broadened our scope by collecting rail eggshells and egg contents. Furthermore, our efforts included extending the sampling period and incorporating samples from spring-fed marshes and from more ag-fed and river-fed marshes surrounding the Salton Sea.

As central-place foragers (Orians & Pearson, 1979), female and male Yuma Ridgway’s rails have relatively small home ranges (Conway et al., 1993) and forage near their nests to minimize their energy expenditure while taking turns incubating eggs and foraging (Eddleman & Conway, 2020). These patterns suggest Yuma Ridgway’s rails nesting and foraging in ag-fed marshes may face greater dietary exposure risk than rails in river-fed or spring-fed marshes (Ricca et al., 2022). All prey species that we captured were introduced to the Salton Sea (USFWS, 2024). It is unknown what proportion of potential rail prey consists of native species, such as the desert pupfish (*Cyprinodon macularius*); however, there are limited data that reported average selenium concentrations of 5.40 ppm (1 carcass) and 4.89 ppm (4 whole body) in pupfish inhabiting irrigation drains and river-fed wetlands surrounding the Salton Sea (Bennett, 1998; Saiki et al., 2012; Miles & Ricca, unpublished data; De La Cruz et al., 2022; Rosen et al., 2023). Crayfish and mosquitofish are known prey items of Yuma Ridgway’s rails (Eddleman, 1989; Ohmart & Tomlinson, 1977) and mosquitofish made up the highest proportion of prey captured in marshes of all three water sources (Online Resource 3-Table 1). Although Yuma Ridgway’s rails are opportunistic foragers (Eddleman & Conway, 2020), their selenium exposure risk is likely influenced by both prey abundance and accessibility. Prey size, for example, may aid in ease of capture. Selenium concentrations are higher in smaller crayfish (< 5 g) than medium (5–15 g) and larger (> 15 g) crayfish in both river-fed and ag-fed marshes at the Salton Sea (Ricca et al., 2022). Additionally, although mosquitofish were the most frequently captured prey in our traps (Online Resource 3-Table 1), rails may consume slower-moving or more easily accessible prey in greater quantities when available. We captured relatively few individuals of species other than mosquitofish and crayfish; however, if sailfin molly, Mozambique tilapia, shrimp, or tadpoles (i.e., prey with the highest average selenium concentrations; Table 1; Online Resource 3-Fig. 2) are slower, more visible, or easier to capture than mosquitofish, they could contribute more to selenium exposure despite being less abundant. Therefore, understanding the interplay between prey availability, ease of capture, and selenium bioaccumulation is essential for assessing dietary exposure risks in different marsh environments.

Yuma Ridgway’s rails at the Salton Sea are primarily non-migratory (although some rails at the Salton Sea do migrate; Harrity & Conway, 2020b) and exhibit high site fidelity between years. The difference in selenium concentrations among blood, breast feather, and head feather samples, particularly of adults, may reflect differences in deposition kinetic pools or differences in temporal uptake. Fast kinetic pools rapidly distribute selenium in the body, making it readily available for various metabolic functions, whereas slow kinetic pools release selenium gradually, serving as long-term reserves (Wastney et al., 1998). Selenium in the blood is part of the fast kinetic pool, but selenium in feathers is part of the slow kinetic pool due to its integration into the keratin structure, making it less accessible for immediate metabolic use (Burger et al., 1994; Couloigner et al., 2015; Wilson et al., 1997).

Temporally, blood samples represent the most recent concentration of selenium in the diet among our samples (Burger et al., 2018). In comparison, feathers reflect selenium in the diet during feather growth (i.e., a two- or three-week window when the sampled feather last molted; Ackerman et al., 2016; Burger & Gochfeld, 1997). Yuma Ridgway’s rail body molt is not temporally constrained and may occur throughout the year. As such, the location and timeframe of selenium deposition into rail body feathers are not explicit and likely vary among individual birds and among individual feathers on the same bird. An adult Yuma Ridgway’s rail may have grown ≥ 1 feather we collected while at a different water source than where we captured that rail. Moreover, selenium concentrations in prey at the Salton Sea may be lower during fall or winter when some body feathers were grown (we only sampled from March to July for all tissue types and all prey). Water selenium concentrations were lower during the winter months in another system with an agricultural "off-season" (Patterson et al., 2010). The Salton Sea supports year-round crop growth and additional studies are needed to determine the extent to which selenium levels vary seasonally at the Salton Sea. The cooler temperatures in winter may lead to reduced evaporation and a decreased need for water by crops. A reduction in water use could result in a lower selenium load, potentially explaining the lower selenium levels found in breast feathers, particularly if those feathers primarily grew during the winter. In 2020, we collected breast feathers without separating grown feathers from actively molting feathers, which may have increased the variation in feather selenium results. In 2021, we only collected fully grown feathers to account for this variation. In addition, we collected head feathers in 2021, a tissue type rarely analyzed for selenium concentration in prior studies. The higher geometric mean of selenium in head feathers compared to breast feathers, a pattern also noted in a previous study on rails (Ricca et al., 2022), suggests that head feathers may better reflect recent habitat use relative to breast feathers (i.e., head feathers likely molted closer to rail capture date than breast feathers).

Slight differences in blood selenium levels between females and males (Table 2, Online Resource 1-Table 1a, 1b) may result from females’ ability to deposit selenium into their eggs (Burger, 1994). This process, combined with females nesting and foraging in ag-fed marshes, could explain the elevated selenium levels observed in egg content and juvenile rail tissues in ag-fed marshes. Beyond this consistent pattern of higher selenium concentrations in ag-fed marshes, juvenile rails also exhibited slightly higher selenium levels than adults (specifically in breast and head feathers). This pattern is likely caused by one or more of the following differences between age classes: 1) feathers of juvenile Yuma Ridgway’s rails were grown more recently and in the marshes near the trapping location, whereas some of the adult feathers we sampled were likely grown elsewhere, 2) dietary differences between the age classes (e.g., prey type, size of prey), or 3) before they attain adult size, juvenile Yuma Ridgway’s rails likely eat more biomass per day to meet the caloric demands of their rapid growth (Eddleman & Conway, 2020; Gochfeld et al., 1996). Juvenile rails likely forage primarily in the vicinity of their nest area initially (Burger & Gochfeld, 1997; Eddleman & Conway, 2020). Therefore, tissue samples from juvenile rails should indicate the exposure to selenium concentrations from the local environment more so than adult tissue samples (Burger & Gochfeld, 1997).

Our study is one of the few to document age-related variation in selenium concentrations in bird tissues. Most prior studies on other species have reported no age-related variation in selenium concentrations of bird feathers (Burger & Gochfeld, 2009; Janssens et al., 2001; Lucia et al., 2010) or bird blood (Burger et al., 2018), but one prior study on gulls reported higher selenium in the feathers of juvenile birds compared to adults (Burger et al., 2009). Age-related differences in selenium levels may be influenced by the age at which juveniles were sampled. One study on kestrels found that one-week-old chicks had lower blood selenium concentrations than their parents (Santolo & Yamamoto, 2009). However, by the second week, some chicks exhibited higher selenium levels than their parents, while others still had lower concentrations, suggesting individual variation in selenium accumulation rates. Another study on kestrels reported that blood selenium levels did not increase significantly beyond five weeks of being fed constant low or high selenium diets (Yamamoto et al., 1998); however, a study on mallards observed a similar plateau effect after 12 weeks of being fed a constant selenium diet (Heinz & Fitzgerald, 1993). These findings indicate that blood selenium concentrations in chicks may vary depending on their age at sampling and the time required for dietary selenium to reach equilibrium in rail diets. Larger sample sizes may be needed to better determine the extent of age-related differences in selenium levels of rails.

The lack of differences in selenium concentration in eggshells among the three water sources may reflect the order of egg formation and the composition of eggshells. Eggshells are the last part of eggs to form within birds before they are laid (Romanoff & Romanoff, 1949) and are approximately 95% calcium carbonate (Shwetha et al., 2018). Selenium in eggshells is mainly a structural component, making the outer layer part of the slow kinetic pool. However, eggshell selenium may also serve as an additional source for embryonic development (Surai et al., 2004). This hypothesis was supported by results from fertile chicken eggs, which revealed a significant decrease in eggshell selenium concentration during embryonic development (Golubkina & Papazyan, 2006). In contrast, selenium in the egg content, which is deposited from the female (Burger, 1994), is more readily available for metabolic functions and is therefore part of the fast kinetic pool. Our mixed effects modeling suggested no difference in egg content selenium concentrations between river-fed and ag-fed marshes even though eggs collected from ag-fed marshes were higher than all but one egg (9.10 ppm) from a river-fed marsh. One study on rails at the Salton Sea in 1990 reported selenium concentrations of 4.98 and 7.75 ppm in two rail eggs collected from river-fed marshes (Roberts, 1996). Selenium values from that study were higher than all egg contents in river-fed marshes from our study besides the 9.10 ppm egg (Roberts, 1996), suggesting that eggs may be similar between the two water sources. The lack of statistical difference may be explained by limited sample size or limited information on parental foraging location prior to nesting.

Some of the variation in selenium concentrations among rail tissues, eggs, and prey likely reflects variation in water dynamics and marsh characteristics. Inflow velocities vary among marshes around the Salton Sea and differ between ag-fed and river-fed marshes. Federal and state management agencies systematically allocate water to river-fed marshes, using cyclical wetting and drying, including drawdowns, to regulate vegetation and promote the health of many wetland-obligate species. However, ag-fed marshes are less predictable, only receiving water if or when farmers are actively irrigating their fields – an increasingly inconsistent process due to the Quantification Settlement Agreement (QSA) and other shifts in Colorado River water allocation that have reduced agricultural water availability in recent years (Coachella Valley Water District et al., 2002). The amount and consistency of water needed in agricultural fields are crop-specific and farmers rotate crops seasonally and may change crops each year. We tested the prediction that selenium concentrations would be lower as marsh inflow velocity increased to account for the variation in inflow velocity. In marshes at the Salton Sea, Yuma Ridgway’s rail tissue, egg, and prey selenium concentrations were primarily negatively correlated with inflow velocity. Selenium concentrations documented in other bird eggs had lower selenium concentrations in flow-through marshes (Davis et al., 2008) and mitigation wetlands (Gordus, 1999) than eggs from nearby ag-fed evaporation basins. Discharge was negatively associated with water selenium concentrations in Wyoming (Patterson et al., 2010) and freshwater inflow rate was negatively associated with selenium concentrations in clams within a tidal estuary in San Francisco Bay (Stewart et al., 2013). Although increased water may lead to higher selenium levels, it may also facilitate more consistent movement of selenium, preventing it from accumulating in one specific area of the marsh. Studies comparing lentic and lotic systems have consistently found higher selenium bioaccumulation in lentic environments, underscoring the need for water quality standards tailored to each system (Adams et al., 2000; Simmons & Wallschlager, 2005). Moreover, marsh size contributed to the variation in selenium concentrations for rail feathers and mosquitofish. Additionally, marsh age (which we were unable to accurately determine in this study) may influence variation in selenium concentrations among aquatic and semi-aquatic species, as older marshes have had more time for selenium to accumulate and persist in the ecosystem. Our mechanistic understanding of these processes would be facilitated by further research that examines changes in selenium concentrations as inflow velocity shifts throughout the year in marshes of different size, age, and water source by routinely resampling both prey and inflow velocity at the same capture locations.

We examined whether selenium concentrations in eggs and prey declined with distance from a marsh’s inflow but found no association between the distance to marsh inflow and selenium concentrations in eggs, mosquitofish, or crayfish. Our results do not align with findings from a previous study, where experimental flow-through wetlands had lower selenium levels at the outflow compared to the inflow (Gao et al., 2000). Gao et al. (2000) also reported that vegetation type influenced selenium retention, with homogenous cattail wetlands showing the second lowest outflow selenium concentrations among eight vegetation types and one open-water plot. While marshes at the Salton Sea are predominantly cattail, distance to inflow may only be a significant factor in smaller marshes or marshes with higher and more consistent inflow. Our results suggest that where Yuma Ridgway’s rails nest and forage in a marsh may be less important than which marsh they nest and forage in. The lack of support for this prediction may be due, in part, to the limited sample size of eggs analyzed and future research with larger sample sizes will help clarify these relationships.

Variation in selenium concentrations among marshes of different water sources, inflow velocities, and size are inconsequential without an understanding of selenium toxicity thresholds. Multiple tissue-based selenium toxicity thresholds have been suggested for several wetland birds, though they differ by species. Some thresholds exist for eggs and tissues of non-threatened birds; however, their applicability across species remains debated due to species-specific variation. Endangered species, like the Yuma Ridgway’s rail may be more susceptible to the effects of selenium due to their restricted geographical distribution and lower population size. The most common dietary selenium toxicity thresholds suggested in the literature are 3–4 ppm (Hamilton, 2004). Based on previous research in lentic ecosystems (Hamilton et al., 1990, unpublished data; Hilton et al., 1980; Lemly, 1995, 1996a, 1996b, 1998, 2002; Lusk, 1993; Rusk, 1991), the USFWS recommended a dietary selenium threshold of 3 ppm to protect fish and aquatic/semiaquatic birds from chronic toxicosis (King et al., 2000; USDOI, 1998). Three Salton Sea studies from 1999, 2008–2009, and 2016 (King et al., 2000; McKernan et al., 2016; Ricca et al., 2022) observed selenium concentrations in agricultural drainage water, fish, and invertebrates that exceeded U.S. Environmental Protection Agency water quality standards for lentic environments (USEPA, 2021). Our results also indicated that many rail prey samples collected at the Salton Sea exceeded the proposed 3 ppm toxicity threshold regardless of water source.

Overall, 53% of egg content samples from river-fed (*n* = 7) and ag-fed (*n* = 8) marshes (2021–2022) exceeded 3 ppm, and 88% of eggshell samples from ag-fed marshes surpassed this threshold. Notably, 27% of all egg contents exceeded 6 ppm, including two eggs from ag-fed marshes and one from a river-fed marsh. Reproductive impairments, including reduced hatchability, embryotoxicity, and teratogenesis, have been documented in multiple avian species at selenium concentrations ≥ 6 ppm in eggs (USDOI, 1998; Skorupa, 1998, 1999; Lam et al., 2005; Beckon et al., 2008; Ohlendorf & Heinz, 2011). A threshold of 3 ppm has been proposed for the onset of teratogenic effects in piscivorous birds (Lemly, 1993). Normal selenium levels considered non-adverse in avian eggs are typically reported as < 3 ppm, with wild bird eggs generally ranging between 1.5–2.5 ppm (Ohlendorf & Harrison, 1986; Skorupa & Ohlendorf, 1991; USDOI, 1998; Eisler, 2000; Schwarzbach et al., 2006; Ohlendorf & Heinz, 2011; Ackerman et al., 2015). Maximum background concentrations have been recorded at ≤ 5 ppm (USDOI, 1998; Ohlendorf & Heinz, 2011).

In selenium-normal environments, the mean concentration in avian blood is between 0.48–1.9 ppm (USDOI, 1998; Eisler, 2000). A selenium toxicity threshold of 4.8 ppm in the blood has been correlated with effects on survival and reproductive success and is often proposed as a threshold for birds (Heinz et al., 1990; Heinz & Fitzgerald, 1993; O’Toole & Raisbeck, 1997; USDOI, 1998; Yamamoto et al., 1998; Santolo et al., 1999; Eisler, 2000) but chronic toxicosis has also been documented in birds when selenium concentrations in blood were > 1 and 2 ppm (McKernan et al., 2016). All rail blood selenium concentrations in our study were above 1 ppm and only one blood sample was below 2 ppm; the majority (96%) of rail blood samples were above 4.8 ppm.

The presumed selenium toxicity thresholds for avian breast feathers vary greatly among studies because feathers reflect selenium in the diet when the feather was growing (Ackerman et al., 2016; Burger & Gochfeld, 1997) but background levels typically range from 1–2 ppm and sometimes from 1–4 ppm (Burger, 1993; Ohlendorf, 1993; USDOI, 1998; Eisler, 2000). All rail breast feather selenium concentrations in our study were above 1 ppm, 90% were above 2 ppm, and 69% were above 4 ppm. Our findings were consistent with a study at the Salton Sea in 2016 that found 100% of rail head feathers and 40% of rail breast feathers above 5 ppm in ag-fed marshes (Ricca et al., 2022). In contrast to our results, Ricca et al. (2022) observed no rail head feathers or breast feathers above 5 ppm in river-fed marshes.

## Conclusion

Selenium bioaccumulation in aquatic food webs likely poses a risk to the survival and reproductive success of wetland-obligate birds like the endangered Yuma Ridgway’s rail. Selenium concentrations at the Salton Sea were highest in ag-fed marshes and marshes with less inflow but were not related to distance to inflow. Yuma Ridgway’s rail tissue, egg, and prey samples collected predominately from ag-fed marshes but also from river-fed marshes surpassed several reported selenium toxicity thresholds. This highlights the importance of monitoring water quality to ensure safe selenium levels in both types of marshes. However, if rails are preferentially foraging and nesting in ag-fed marshes (the predominant wetland type available at the Salton Sea), they may be at risk of an ecological trap. Wetlands surrounding the Salton Sea support one of the two remaining core Yuma Ridgway’s rail population areas in the U.S. The health of these wetlands is likely vital for the success and delisting of the Yuma Ridgway’s rail. Our results suggest that increased input of Colorado River water or spring water into ag-fed marshes that support Yuma Ridgway’s rails may reduce the dietary risk of selenium bioaccumulation to rails. Management practices could focus on restoring or enhancing ag-fed marshes to provide optimal nesting habitats for the rails by exploring other methods such as improving the quality of agricultural drainage water through filtration or treatment.

## Supplementary information

Below are the links to the electronic supplementary materials.

Online Resource 1 (PDF 353 KB)

Online Resource 2 (PDF 195 MB)

Online Resource 3 (PDF 610 KB)

## Data Availability

No datasets were generated or analysed during the current study.

## References

[CR1] Ackerman, J. T., Eagles-Smith, C. A., Herzog, M. P., & Hartman, C. A. (2016). Maternal transfer of contaminants in birds: Mercury and selenium concentrations in parents and their eggs. *Environmental Pollution,**210*, 145–154. 10.1016/j.envpol.2015.12.016 26708769 10.1016/j.envpol.2015.12.016

[CR2] Ackerman, J. T., Herzog, M. P., Hartman, C. A., Isanhart, J., Herring, G., Vaughn, S., Cavitt, J. F., Eagles-Smith, C. A., Browers, H., Cline, C., & Vest, J. (2015). Mercury and selenium contamination in waterbird eggs and risk to avian reproduction at Great Salt Lake, Utah. (Open-File Report 2015–1020) U.S. Geological Survey. 10.3133/ofr20151020

[CR3] Adams, W. J, Toll, J. E., Brix, K. V., Tear, L. M., & DeForest, D. K. (2000). Site-specific approach for settling water quality criteria for selenium: Differences between lotic and lentic systems. Proceedings Mine Reclamation Symposium: Selenium Session; Sponsored by Ministry of Energy and Mines, Williams Lake, British Columbia, Canada, June 21–22, 2000. 10.14288/1.0042374

[CR4] Barnum, D. A., Bradley, T., Cohen, M., Wilcox, B., & Yanega, G. (2017). State of the Salton Sea – A science and monitoring meeting of scientists for the Salton Sea. (Open-File Report 2017–1005) U.S. Geological Survey. 10.3133/ofr20171005

[CR5] Bates, D., Mächler, M., Bolker, B., & Walker, S. (2015). Fitting linear mixed-effects models using lme4. *Journal of Statistical Software,**67*(1), 1–48. 10.18637/jss.v067.i01

[CR6] Beckon, W. N., Parkins, C., Maximovich, A., & Beckon, A. V. (2008). A general approach to modeling biphasic relationships. *Environmental Science & Technology,**42*(4), 1308–1314. 10.1021/es071148m 18351110 10.1021/es071148m

[CR7] Bennett, J. (1998). *Biological effects of selenium and other contaminants associated with irrigation drainage in the Salton Sea area, California 1992–1994.* Department of the Interior, National Irrigation Water Quality Program Information Report No. 4. Available from https://nrm.dfg.ca.gov/FileHandler.ashx?DocumentID=8734. Accessed 22 Oct 2024.

[CR8] Bruehler, G., & de Peyster, A. (1999). Selenium and other trace metals in pelicans dying at the Salton Sea. *Bulletin of Environmental Contamination and Toxicology,**63*, 590–597. 10.1007/s001289901021 10541677 10.1007/s001289901021

[CR9] Burger, J. (1993). Metals in avian feathers: Bioindicators of environmental pollution. *Reviews in Environmental Toxicology,**5*, 197–306.

[CR10] Burger, J. (1994). Heavy metals in avian eggshells: Another excretion method. *Journal of Toxicology and Environmental Health, Part A Current Issues,**41*(2), 207–220. 10.1080/15287399409531837 10.1080/15287399409531837 8301699

[CR11] Burger, J., & Gochfeld, M. (1997). Risk, mercury levels, and birds: Relating adverse laboratory effects to field biomonitoring. *Environmental Research,**75*(2), 160–172. 10.1006/enrs.1997.3778 9417847 10.1006/enrs.1997.3778

[CR12] Burger, J., & Gochfeld, M. (2009). Comparison of arsenic, cadmium, chromium, lead, manganese, mercury and selenium in feathers in bald eagle (*Haliaeetus leucocephalus*), and comparison with common eider (*Somateria mollissima*), glaucous-winged gull (*Larus glaucescens*), pigeon guillemot (*Cepphus columba*), and tufted puffin (*Fratercula cirrhata*) from the Aleutian Chain of Alaska. *Environmental Monitoring and Assessment,**152*, 357–367. 10.1007/s10661-008-0321-7 18521716 10.1007/s10661-008-0321-7 PMC4300136

[CR13] Burger, J., Gochfeld, M., Jeitner, C., Burke, S., Volz, C. D., Snigaroff, R., Snigarof, D., Shukla, T., & Shukla, S. (2009). Mercury and other metals in eggs and feathers of glaucous-winged gulls (*Larus glaucescens*) in the Aleutians. *Environmental Monitoring and Assessment,**152*, 179–194. 10.1007/s10661-008-0306-6 18626778 10.1007/s10661-008-0306-6 PMC4300123

[CR14] Burger, J., Mizrahi, D., Tsipoura, N., Jeitner, C., & Gochfeld, M. (2018). Mercury, lead, cadmium, cobalt, arsenic and selenium in the blood of semipalmated sandpipers (*Calidris pusilla*) from Suriname, South America: Age-related differences in wintering site and comparisons with a stopover site in New Jersey, USA. *Toxics,**6*(2), Article 27. 10.3390/toxics6020027 29747411 10.3390/toxics6020027 PMC6027228

[CR15] Burger, J., Nisbet, I. C. T., & Gochfeld, M. (1994). Heavy metal and selenium levels in feathers of known-aged common terns (*Sterna hirundo*). *Archives of Environmental Contamination and Toxicology,**26*, 351–355. 10.1007/BF00203562 8161233 10.1007/BF00203562

[CR16] Burnham, K. P., & Anderson, D. R. (2002). *Model selection and multi-model inference: A practical information-theoretic approach* (2nd ed.). Springer. 10.1007/b97636

[CR17] Byron, E. R., & Ohlendorf, H. M. (2007). Diffusive flux of selenium between lake sediment and overlying water – Assessing restoration alternatives for the Salton Sea. *Lake and Reservoir Management,**23*(5), 630–636. 10.1080/07438140709354042

[CR18] California Department of Food and Agriculture. (2017). Planting seeds blog. California Department of Food and Agriculture. Available from https://plantingseedsblog.cdfa.ca.gov/wordpress/?p=13929. Accessed 30 July 2024.

[CR19] Coachella Valley Water District, Imperial Irrigation District, Metropolitan Water District of Southern California, & San Diego County Water Authority. (2002). *Draft program environmental impact report for the implementation of the Colorado river quantification settlement agreement*: State clearinghouse no. 2000061034, v. I and II. Available from https://www.waterboards.ca.gov/waterrights/water_issues/programs/hearings/iid_sdcwa/iid/exhibits/iid56.pdf. Accessed 19 Aug 2023.

[CR20] Conway, C. J. (2011). Standardized North American marsh bird monitoring protocol. *Waterbirds,**34*(3), 319–346. 10.1675/063.034.0307

[CR21] Conway, C. J., & Eddleman, W. R. (2000). Yuma clapper rail. In R. P. Reading, & B. J. Miller (Eds.), *Endangered animals: A reference guide to conflicting issues* (pp. 277–284). Greenwood Press.

[CR22] Conway, C. J., Eddleman, W. R., Anderson, S. H., & Hanebury, L. R. (1993). Seasonal changes in Yuma clapper rail vocalization rate and habitat use. *The Journal of Wildlife Management,**57*(2), 282–290. 10.2307/3809425

[CR23] Conway, C. J., Nadeau, C. P., & Piest, L. (2010). Fire helps restore natural disturbance regime to benefit rare and endangered marsh birds endemic to the Colorado River. *Ecological Applications,**20*(7), 2024–2035. 10.1890/09-1624.1 21049887 10.1890/09-1624.1

[CR24] Couloigner, F., Jlali, M., Briens, M., Rouffineau, F., Geraert, P. A., & Mercier, Y. (2015). Selenium deposition kinetics of different selenium sources in muscle and feathers of broilers. *Poultry Science,**94*(11), 2708–2714. 10.3382/ps/pev282 10.3382/ps/pev282 26500270

[CR25] Davis, D. E., Hanson, C. H., & Hansen, R. B. (2008). Constructed wetland habitat for American avocet and black-necked stilt foraging and nesting. *The Journal of Wildlife Management,**72*(1), 143–151. 10.2193/2005-553

[CR26] De La Cruz, S. E. W., Woo, I., Antonino, C. Y., Hall, L. A., Ricca, M. A., & Miles, A. K. (2022). Biological tissue data used to evaluate selenium hazards in the Salton Sea ecosystem (1984–2020). (Data release) U.S. Geological Survey. 10.5066/P9ECP7O0

[CR27] Dwyer, T. J. (1972). An adjustable radio package for ducks. *Bird-Banding,**43*(4), 282–284. 10.2307/4511905

[CR28] Eddleman, W. R. (1989). Biology of the Yuma clapper rail in the Southwestern U.S. and Northwestern Mexico final report. (Fish and Wildlife Service Contract 4-AA-30–02060) Wyoming Cooperative Research Unit, University of Wyoming.

[CR29] Eddleman, W. R., & Conway, C. J. (2020). Ridgway’s rail *(Rallus obsoletus)*. Birds of the World. Available from https://birdsoftheworld.org/bow/species/ridrai1/cur/introduction. Accessed 16 Oct 2024.

[CR30] Eisler, R. (2000). Selenium. In R. Eisler (Ed.), *Handbook of chemical risk assessment: Health hazards to humans, plants, and animals* (Vol. 3., pp. 1649–1705). Lewis Publishers.

[CR31] Environmental Systems Research Institute [ESRI]. (2010). USA national atlas water feature lines rivers and streams [Layer Package]. Available from https://www.arcgis.com/home/item.html?id=8206e517c2264bb39b4a0780462d5be1. Accessed 31 Oct 2024.

[CR32] Environmental Systems Research Institute [ESRI]. (2024). ArcGIS Pro (Version 3.2.0) [Software]. Environmental Systems Research Institute. Available from https://www.esri.com. Accessed 15 Feb 2024.

[CR33] Gao, S., Tanji, K. K., Peters, D. W., & Herbel, M. J. (2000). Water selenium and sediment fractionation in a California flow-through wetland system. *Journal of Environmental Quality,**29*(4), 1275–1283. 10.2134/jeq2000.00472425002900040034x

[CR34] Garone, P. (1999). The tragedy at Kesterson Reservoir: A case study in environmental history and a lesson in ecological complexity. *Environs: Environmental Law and Policy Journal,**22*(2), 107–144.

[CR35] Gochfeld, M., Belant, J. L., Shukla, T., Benson, T., & Burger, J. (1996). Heavy metals in laughing gulls: Gender, age, and tissue differences. *Environmental Toxicology and Chemistry,**15*(12), 2275–2283. 10.1002/etc.5620151223

[CR36] Golubkina, N. A., & Papazyan, T. T. (2006). Selenium distribution in eggs of avian species. *Comparative Biochemistry and Physiology, Part B: Biochemistry & Molecular Biology,**145*(3–4), 384–388. 10.1016/j.cbpb.2006.08.007 10.1016/j.cbpb.2006.08.007 17055312

[CR37] Gordus, A. G. (1999). Selenium concentrations in eggs of American avocets and black-necked stilts at an evaporation basin and freshwater wetland in California. *The Journal of Wildlife Management,**63*(2), 497–501. 10.2307/3802634

[CR38] Griffiths, R., Double, M. C., Orr, K., & Dawson, R. J. G. (1998). A DNA test to sex most birds. *Molecular Ecology,**7*(8), 1071–1075. 10.1046/j.1365-294x.1998.00389.x 9711866 10.1046/j.1365-294x.1998.00389.x

[CR39] Hamilton, S. J. (2004). Review of selenium toxicity in the aquatic food chain. *Science of the Total Environment,**326*(1–3), 1–31. 10.1016/j.scitotenv.2004.01.019 15142762 10.1016/j.scitotenv.2004.01.019

[CR40] Hamilton, S. J., Buhl, K. J., Faerber, N. L., Wiedmeyer, R. H., & Bullard, F. A. (1990). Toxicity of organic selenium in the diet to chinook salmon. *Environmental Toxicology and Chemistry,**9*(3), 347–358. 10.1002/etc.5620090310

[CR41] Harrity, E. J., & Conway, C. J. (2020). Noose carpets: A novel method to capture rails. *Wildlife Society Bulletin,**44*(1), 15–22. 10.1002/wsb.1068

[CR42] Harrity, E. J., & Conway, C. J. (2020). Satellite transmitters reveal previously unknown migratory behavior and wintering locations of Yuma Ridgway’s rails. *Journal of Field Ornithology,**91*(3), 300–312. 10.1111/jofo.12344

[CR43] Harrity, E. J., Michael, L. E., & Conway, C. J. (2021). Sexual dimorphism in morphology and plumage of endangered Yuma Ridgway’s rails: A model for documenting sex. *Journal of Fish and Wildlife Management,**12*(2), 464–474. 10.3996/JFWM-20-095

[CR44] Harrity, E. J., Stevens, B. S., & Conway, C. J. (2020). Keeping up with the times: Mapping range-wide habitat suitability for endangered species in a changing environment. *Biological Conservation,**250*, Article 108734. 10.1016/j.biocon.2020.108734

[CR45] Heinz, G. H., & Fitzgerald, M. A. (1993). Overwinter survival of mallards fed selenium. *Archives of Environmental Contamination and Toxicology,**25*, 90–94. 10.1007/BF00230717

[CR46] Heinz, G. H., Pendleton, G. W., Krynitsky, A. J., & Gold, L. G. (1990). Selenium accumulation and elimination in mallards. *Archives of Environmental Contamination and Toxicology,**19*, 374–379. 10.1007/BF01054981 2353836 10.1007/BF01054981

[CR47] Hilton, J. W., Hodson, P. V., & Slinger, S. J. (1980). The requirement and toxicity of selenium in rainbow trout (*Salmo gairdneri*). *The Journal of Nutrition,**110*(12), 2527–2535. 10.1093/jn/110.12.2527 7441379 10.1093/jn/110.12.2527

[CR48] Imperial County Agricultural Commission. (2020). 2019 Imperial county agricultural crop and livestock Report*. *Available from https://agcom.imperialcounty.org/wp-content/uploads/2020/12/2019-Crop-Report.pdf. Accessed 16 Oct 2024.

[CR49] Imperial County Agricultural Commission. (2021). 2020 Imperial county agricultural crop and livestock report. Available from https://agcom.imperialcounty.org/wp-content/uploads/2021/08/2020-Crop-Report-v2.pdf. Accessed 16 Oct 2024.

[CR50] Imperial County Agricultural Commission. (2022). 2021 Imperial county agricultural crop and livestock report. Available from https://agcom.imperialcounty.org/wp-content/uploads/2022/10/2021-CR-Draft-Final.pdf. Accessed 16 Oct 2024.

[CR51] Imperial County Agricultural Commission. (2023). 2022 Imperial county agricultural crop and livestock report. Available from https://agcom.imperialcounty.org/wp-content/uploads/2023/10/2022-Crop-Report-Updated.pdf. Accessed 16 Oct 2024.

[CR52] Imperial Irrigation District. (2024). IID ‘Water’ homepage. Available from https://www.iid.com/water/water-transportation-system/colorado-river-facilities/all-american-canal. Accessed 9 Aug 2024.

[CR53] Janssens, E., Dauwe, T., Bervoets, L., & Eens, M. (2001). Heavy metals and selenium in feathers of great tits (*Parus major*) along a pollution gradient. *Environmental Toxicology and Chemistry,**20*(12), 2815–2820. 10.1002/etc.5620201221 11764165

[CR54] Kaufmann, G. W. (1989). Breeding ecology of the sora *Porzana carolina*, and the Virginia rail *Rallus limicola*. *Canadian Field-Naturalist,**103*, 270–282.

[CR55] Kimbrough, K. L., & Lauenstein, G. G. (2006). *Major and trace element analytical methods of the national status and trends program*: 2000–2006. (Technical Memorandum NOS NCCOS 29) National Oceanic and Atmospheric Administrations. Available from https://repository.library.noaa.gov/view/noaa/17784. Accessed 19 Aug 2023.

[CR56] King, K. A., Velasco, A. L., Garcia-Hernandez, J., Zaun, B. J., Record, J., & Wesley, J. (2000). Contaminants in potential prey of the Yuma clapper rail: Arizona and California, USA, and Sonora and Baja, Mexico, 1998–1999.U.S. Fish and Wildlife Service, Arizona Ecological Services Office – Contaminants Program*. *Available from https://azmemory.azlibrary.gov/nodes/view/207622. Accessed 23 May 2024.

[CR57] Kitowski, I., Jakubas, D., Indykiewicz, P., & Dariusz, W. (2018). Factors affecting element concentrations in eggshells of three sympatrically nesting waterbirds in Northern Poland. *Archives of Environmental Contamination and Toxicology,**74*, 318–329. 10.1007/s00244-017-0481-y 29170796 10.1007/s00244-017-0481-y PMC5807457

[CR58] Lam, J. C. W., Tanabe, S., Lam, M. H., & Lam, P. K. S. (2005). Risk to breeding success of waterbirds by contaminants in Hong Kong: Evidence from trace elements in eggs. *Environmental Pollution,**135*(3), 481–490. 10.1016/j.envpol.2004.11.021 15749545 10.1016/j.envpol.2004.11.021

[CR59] Lemly, A. D. (1993). Guidelines for evaluating selenium data from aquatic monitoring and assessment studies. *Environmental Monitoring and Assessment,**28*, 83–100. 10.1007/BF00547213 24221061 10.1007/BF00547213

[CR60] Lemly, A. D. (1995). A protocol for aquatic hazard assessment of selenium. *Ecotoxicology and Environmental Safety,**32*(3), 280–288. 10.1006/eesa.1995.1115 8964256 10.1006/eesa.1995.1115

[CR61] Lemly, A. D. (1996). Assessing the toxic threat of selenium to fish and aquatic birds. *Environmental Monitoring and Assessment,**43*, 19–35. 10.1007/BF00399568 24193731 10.1007/BF00399568

[CR62] Lemly, A. D. (1996b). Selenium in aquatic organisms. In W. N. Beyer, G. H. Heinz, & A. W. Redmon-Norwood (Eds.), *Environmental contaminants in wildlife: Interpreting tissue concentrations* (pp. 427–445). CRC Press.

[CR63] Lemly, A. D. (1998). Pathology of selenium poisoning in fish. In W. T. Frankenberger & R. A. Engberg (Eds.), *Environmental chemistry of selenium* (pp. 281–296). Marcel Dekker Inc.

[CR64] Lemly, A. D. (2002). *Selenium assessment in aquatic ecosystems: A guide for hazard evaluation and water quality criteria*. Springer-Verlad New York Inc.

[CR65] Lucia, M., André, J., Gontier, K., Diot, N., Veiga, J., & Davail, S. (2010). Trace element concentrations (mercury, cadmium, copper, zinc, lead, aluminum, nickel, arsenic, and selenium) in some aquatic birds of the southwest Atlantic coast of France. *Archives of Environmental Contamination and Toxicology,**58*, 844–853. 10.1007/s00244-009-9393-9 19763676 10.1007/s00244-009-9393-9

[CR66] Lusk, J. D. (1993). *Selenium in aquatic habitats at imperial national wildlife refuge*. Cooperative Fish and Wildlife Research Unit, University of Arizona. Thesis. Available from https://www.geo.arizona.edu/rcncrd/documents/Lusk_1993.pdf. Accessed 4 July 2023.

[CR67] McCarthy, K. A., & Johnson, H. M. (2009). Effect of agricultural practices on hydrology and water chemistry in a small irrigated catchment, Yakima River Basin, Washington. (Scientific Investigations Report 2009–5030) U.S. Geological Survey. 10.3133/sir20095030

[CR68] McKernan, M. A., Reynolds, K. D., Zeeman, C. T., & Takekawa, J. Y. (2016). Selenium exposure of Yuma clapper rails (*Rallus longirostris yumanensis*) inhabiting the Lower Colorado River and the Salton Sea. U.S. Fish and Wildlife Service – Environmental Contaminants Program.

[CR69] National Oceanic and Atmospheric Administration [NOAA]. (2024). National centers for environmental information internet services team. Past weather for the imperial county airport, California station. Available from https://www.ncei.noaa.gov/access/past-weather/Imperial%20County%20Airport. Accessed 12 Sept 2024.

[CR70] Norton, C. L., Dannenberg, M. P., Yan, D., Wallace, C. S. A., Rodriguez, J. R., Munson, S. M., van Leeuwen, W. J. D., & Smith, W. K. (2021). Climate and socioeconomic factors drive irrigated agriculture dynamics in the Lower Colorado River Basin. *Remote Sensing,**13*(9), Article 1659. 10.3390/rs13091659

[CR71] O’Toole, D., & Raisbeck, M. F. (1997). Experimentally induced selenosis of adult mallard ducks: Clinical signs, lesions, and toxicology. *Veterinary Pathology,**34*, 330–340. 10.1177/030098589703400409 9240842 10.1177/030098589703400409

[CR72] Ohlendorf, H. M. (1993). Marine birds and trace elements in the temperate North Pacific. In K. Vermeer, K. T. Briggs, K. H. Morgan, & D. Siegel-Causey (Eds.), *The status, ecology, and conservation of marine birds of the North Pacific* (pp. 232–240). Special Publication.

[CR73] Ohlendorf, H. M. (2002). The birds of Kesterson Reservoir: A historical perspective. *Aquatic Toxicology,**57*(1–2), 1–10. 10.1016/S0166-445X(01)00266-1 11879934 10.1016/s0166-445x(01)00266-1

[CR74] Ohlendorf, H. M. (2003). Ecotoxicology of selenium. In D. J. Hoffman, B. A. Rattner, G. A. Burton Jr., & J. Cairns Jr. (Eds.), *Handbook of ecotoxicology* (2nd ed., pp. 465–500). Lewis Publishers. 10.1201/9781420032505

[CR75] Ohlendorf, H. M. (2011). Selenium, salty water, and deformed birds. In J. Elliott, C. Bishop, & C. Morrissey (Eds.), *Wildlife ecotoxicology. Emerging topics in ecotoxicology* (vol. 3, pp. 325–357). Springer. 10.1007/978-0-387-89432-4_11

[CR76] Ohlendorf, H. M., & Harrison, C. S. (1986). Mercury, selenium, cadmium, and organochlorines in eggs of three Hawaiian seabird species. *Environmental Pollution Series b, Chemical and Physical,**11*(3), 169–191. 10.1016/0143-148X(86)90022-4

[CR77] Ohlendorf, H. M., & Heinz, G. H. (2011). Selenium in birds. In W. N. Beyer, & J. P. Meador (Eds.), *Environmental contaminants in biota: Interpreting tissue concentrations* (2nd ed., pp. 669–701). CRC Press.

[CR78] Ohlendorf, H. M., Santolo, G. M., Byron, E. R., & Eisert, M. A. (2020). Kesterson reservoir: 30 years of selenium risk assessment and management. *Integrated Environmental Assessment and Management,**16*(2), 257–268. 10.1002/ieam.4222 31646761 10.1002/ieam.4222

[CR79] Ohmart, R. D., & Tomlinson, R. E. (1977). Foods of western clapper rails. *The Wilson Bulletin,**89*(2), 332–336.

[CR80] Orians, G. H., & Pearson, N. E. (1979). On the theory of central place foraging. In D. J. Horn, R. D. Mitchell, & G. R. Stairs (Eds.), *Analysis of ecological systems* (pp. 154–177). Ohio State University Press.

[CR81] Patterson, M. M., Paige, G. B., & Reddy, K. J. (2010). Selenium in surface and irrigation water in the Kendrick irrigation district, Wyoming. *Environmental Monitoring and Assessment,**171*, 267–280. 10.1007/s10661-009-1277-y 20020321 10.1007/s10661-009-1277-y

[CR82] Popkin, R. (1986). Kesterson: Nonpoint nightmare. *EPA Journal,**12*, 13–14.

[CR83] R Core Team. (2024). R: A language and environment for statistical computing. *R Foundation for Statistical Computing*. Vienna, Austria. Available from http://www.R-project.org/. Accessed 1 May 2024.

[CR84] Ricca, M. A., Overton, C. T., Anderson, T. W., Merritt, A., Harrity, E. J., Matchett, E., & Casazza, M. L. (2022). Yuma Ridgway’s rail selenium exposure and occupancy within managed and unmanaged emergent marshes at the Salton Sea. (Open-File Report 2022–1045) U.S. Geological Survey. 10.3133/ofr20221045

[CR85] Roberts, C. A. (1996). Trace element and organochlorine contamination in prey and habitat of the Yuma Clapper Rail in the Imperial Valley, California. *U.S. Fish and Wildlife Report*. Available from https://nrm.dfg.ca.gov/FileHandler.ashx?DocumentID=7412. Accessed 16 Oct 2024.

[CR86] Romanoff, A. L., & Romanoff, A. J. (1949). *The avian egg*. John Wiley and Sons.

[CR87] Rosen, M. R., De La Cruz, S. E. W., Groover, K. D., Woo, I., Roberts, S. A., Davis, M. J., & Antonio, C. Y. (2023). *Selenium hazards in the Salton Sea environment - Summary of current knowledge to inform future wetland management*. (Scientific Investigations Report 2023–5042) U.S. Geological Survey. 10.3133/sir20235042

[CR88] Rusk, M. K. (1991). *Selenium risk to Yuma clapper rails and other marsh birds of the Lower Colorado River*. Cooperative Fish and Wildlife Research Unit, University of Arizona.

[CR89] Saiki, M. K., Martin, B. A., & May, T. W. (2012). Assessment of two nonnative poeciliid fishes for monitoring selenium exposure in the endangered desert pupfish. *Water, Air, and Soil Pollution,**223*(4), 1671–1683. 10.1007/s11270-011-0974-7

[CR90] Santolo, G. M., & Yamamoto, J. T. (2009). Nest box and site use by, and selenium concentrations in, American kestrels at Kesterson Reservoir. *Central California. Journal of Raptor Research,**43*(4), 315–324. 10.3356/JRR-08-47.1

[CR91] Santolo, G. M., Yamamoto, J. T., Pisenti, J. M., & Wilson, B. W. (1999). Selenium accumulation and effects on reproduction in captive American kestrels fed selenomethionine. *The Journal of Wildlife Management,**63*(2), 502–511. 10.2307/3802635

[CR92] Schwarzbach, S. E., Albertson, J. D., & Thomas, C. M. (2006). Effects of predation, flooding, and contamination on reproductive success of California clapper rails (*Rallus longirostris obsoletus*) in San Francisco Bay. *The Auk,**123*(1), 45–60. 10.1093/auk/123.1.45

[CR93] Seiler, R. L., Skorupa, J. P., Naftz, D. L., & Nolan, B. T. (2003). Irrigation-induced contamination of water, sediment, and biota in the western United States – Synthesis of data from the National Irrigation Water Quality Program. (Professional Paper 1655) U.S. Geological Survey. 10.3133/pp1655

[CR94] Setmire, J. G., & Schroeder, R. A. (1998). Selenium and salinity concerns in the Salton Sea area of California. In W. T. Frankenberger & R. A. Engberg (Eds.), *Environmental chemistry of selenium* (pp. 205–221). Marcel Dekker Inc.

[CR95] Shwetha, A., Dhananjaya, K. S. M., & Ananda, S. M. (2018). Comparative study on calcium content in eggshells of different birds. *International Journal of Zoology Studies,**3*(4), 31–33.

[CR96] Simmons, D. B. D., & Wallschlager, D. (2005). A critical review of the biogeochemistry and ecotoxicology of selenium in lotic and lentic environments. *Environmental Toxicology and Chemistry,**24*(6), 1331–1343. 10.1897/04-176R.1 16117108 10.1897/04-176r.1

[CR97] Skorupa, J. P. (1998). Selenium poisoning of fish and wildlife in nature: Lessons from twelve real world experiences. In W. T. Frankenberger, & R. A. Engberg (Eds.), *Environmental chemistry of selenium* (pp. 315–354). Marcel Dekker Inc.

[CR98] Skorupa, J. P. (1999). Beware of missing data and undernourished statistical models: Comment on Fairbrother et al.’s critical evaluation. *Human Ecology and Risk Assessment,**5*(6), 1255–1262. 10.1080/10807039.1999.10518887

[CR99] Skorupa, J. P., & Ohlendorf, H. M. (1991). Contaminants in drainage water and avian risk thresholds. In A. Dinar & D. Zilberman (Eds.), *The economics and management of water and drainage in agriculture* (pp. 347–368). Springer.

[CR100] Stewart, A. R., Luoma, S. N., Elrick, K. A., Carter, J. L., & van der Wegen, M. (2013). Influence of estuarine processes on spatiotemporal variation in bioavailable selenium. *Marine Ecology Progress Series,**492*, 41–56. 10.3354/meps10503

[CR101] Surai, P. F., Karadas, F., Pappas, A. C., & Dvorska, J. E. (2004). Selenium distribution in eggs of ISA Brown commercial layers. Proceedings of the 20th Annual Symposium “Nutritional Biotechnology in the Feed and Food Industries”, Lexington, USA.

[CR102] U.S. Department of the Interior [USDOI]. (1998). Guidelines for the interpretation of the biological effects of selected constituents in biota, water, and sediment. (Information Report No. 3) National Irrigation Water Quality Program. Available from https://clu-in.org/download/contaminantfocus/arsenic/dept_interior_guidelines.pdf. Accessed 8 Dec 2023.

[CR103] U.S. Environmental Protection Agency [USEPA]. (2021). 2021 Revision - Aquatic life ambient water quality criterion for selenium – freshwater 2016. U.S. Environmental protection agency: Water quality criteria. Available from https://www.epa.gov/wqc/aquatic-life-criterion-selenium. Accessed 1 Dec 2023.

[CR104] U.S. Fish and Wildlife Service [USFWS]. (1967). Office of the secretary native fish and wildlife endangered species*.* Federal Register, 32, 4001. Available from https://www.fws.gov/species-publication-action/endangered-species-list-1967-2. Accessed 14 Oct 2024.

[CR105] U.S. Fish and Wildlife Service [USFWS]. (2010, February 10). Y*uma *clapper rail* (Rallus longirostris yumanensis) *recovery plan. Draft first revision. U.S. Fish and Wildlife Service. Available from https://www.fws.gov/species-publication-action/draft-yuma-clapper-rail-rallus-longirostris-yumanensis-recovery-plan. Accessed 1 May 2024.

[CR106] U.S. Fish and Wildlife Service [USFWS]. (2024). *Sonny Bono Salton Sea National Wildlife Refuge: Species*. U.S. Fish and Wildlife Service. Available from https://www.fws.gov/refuge/sonny-bono-salton-sea/species. Accessed 12 Nov 2024.

[CR107] Wastney, M. E., Patterson, B. H., Linares, O. A., Greif, P. C., & Boston, R. C. (1998). *Investigating biological systems using modeling: Strategies and software*. Academic Press.

[CR108] Wilson, D. S., Zhang, P., He, R., Ota, R., & Omaye, S. T. (1997). Kinetics of selenium incorporation into tissues of female mallard ducks. *Toxicology,**122*(1–2), 51–60. 10.1016/S0300-483X(97)00077-2 9274801 10.1016/s0300-483x(97)00077-2

[CR109] Yamamoto, J. T., Santolo, G. M., & Wilson, B. W. (1998). Selenium accumulation in captive American kestrels (*Falco sparverius*) fed selenomethionine and naturally incorporated selenium. *Environmental Toxicology and Chemistry,**17*(12), 2494–2497. 10.1002/etc.5620171216

