# Supplementary material for: Irrigated agriculture influences selenium levels in an endangered marsh bird
Source: Environ Monit Assess. 2025 Sep 24;197(10):1142. doi: 10.1007/s10661-025-14533-1 (PMC12460551; doi:10.1007/s10661-025-14533-1)
Supplement: Supplementary file 1 — Online Resource 1 (PDF 353 KB) [file 10661_2025_14533_MOESM1_ESM.pdf]

Online Resource 1

Irrigated agriculture influences selenium levels in an endangered marsh bird

Environmental Monitoring and Assessment

Cydney M. Yost, Kathryn M. Sliwa, Razia Shafique-Sabir, Jonathan Shore, & Courtney J. Conway

Cydney M. Yost (corresponding author), Idaho Cooperative Fish & Wildlife Research Unit, University of Idaho, Department of Fish & Wildlife Sciences, Moscow, ID, USA, e-mail: cydney.yost@gmail.com

Supplementary tables, figures, and raw data related to Yuma Ridgway's rail blood, breast feathers, and head feathers

Table 1a Factors that explain variation in selenium concentrations of Yuma Ridgway's rail blood collected from the Salton Sea, California, USA (2020–2021). Phase 1 includes all models with $\Delta AIC_C \leq 2$, the null model, and the global model (sex*mass + age*mass + (1|year)). Phase 2 includes all models with $\Delta AIC_C \leq 2$, the informed null (top model from phase 1), and the global model (sex + age + water source + velocity*marsh size + (1|year)). ΔAIC_C = the difference in Akaike Information Criterion for small sample sizes from the best fitting model; w_i = Akaike weight of the model. Potential explanatory variables included: age (juveniles vs adult); water source (spring-fed, river-fed, or ag-fed); velocity (two-week rolling average of the inflow velocity at the time of rail capture); marsh size (total hectares of continuous cattail marsh)

**Phase 1**		
**Model**	**ΔAIC_C**	**w_i**
sex + age	0.00	0.28
age	0.09	0.27
sex	0.39	0.23
null	0.51	0.22
global	19.52	0.00
**Phase 2**		
sex + age + water source + velocity	0.00	0.97
global	14.45	0.00
informed null	29.35	0.00

Table 1b Parameter estimates (Beta), standard errors (SE), and 95% confidence intervals for all predictor variables included in the top model (phase 2) to explain variation in selenium concentration of Yuma Ridgway's rail blood collected from the Salton Sea, California, USA (2020–2021). Age (adult [reference category] or juvenile); sex (female [reference category] or male); water source (spring-fed, river-fed, or ag-fed [reference category]); velocity (two-week rolling average of the inflow velocity at the time of rail capture)

**Parameter**	**Beta**	**SE**	**Lower 95% CI**	**Upper 95% CI**
intercept	14.11	1.13	11.94	16.28
sex	0.73	0.91	-1.03	2.49
age	-1.42	1.22	-3.77	0.94
water source: river-fed	-5.15	1.02	-7.11	-3.20
water source: spring-fed	-6.97	2.00	-10.83	-3.12
velocity	-9.32	4.38	-17.75	-0.90

Table 2a Factors that explain variation in selenium concentration of Yuma Ridgway's rail breast feathers collected from the Salton Sea, California, USA (2020–2021). Phase 1 includes all models with $\Delta AIC_C \leq 2$, the null model, and the global model (sex*mass + age*mass + (1|year)). Phase 2 includes all models with $\Delta AIC_C \leq 2$, the informed null (top model from phase 1), and the global model (age + water source + velocity*marsh size + (1|year)). ΔAIC_C = the difference in Akaike Information Criterion for small sample sizes from the best fitting model; w_i = Akaike weight of the model. Potential explanatory variables include: age (juvenile vs adult); water source (spring-fed, river-fed, or ag-fed); velocity (two-week rolling average of the inflow velocity at the time of rail capture); marsh size (total hectares of continuous cattail marsh)

Phase 1		
**Model**	**ΔAIC_C**	**w_i**
age	0.00	0.55
sex + age	0.71	0.39
null	10.44	0.00
global	13.91	0.00
**Phase 2**		
age + water source + velocity	0.00	0.42
age + water source + marsh size	0.49	0.33
age + water source	1.69	0.18
global	3.89	0.06
informed null	14.15	0.00

Table 2b Parameter estimates (Beta), standard errors (SE), and 95% confidence intervals for all predictor variables included in the top model (phase 2) to explain variation in selenium concentration of Yuma Ridgway's rail breast feathers collected from the Salton Sea, California, USA (2020–2021). Age (adult [reference category] or juvenile); water source (spring-fed, river-fed, or ag-fed [reference category]); velocity (two-week rolling average of the inflow velocity at the time of rail capture)

Parameter	Beta	SE	Lower 95% CI	Upper 95% CI
intercept	6.44	1.58	2.78	10.10
age	2.76	0.82	1.11	4.33
water source: river-fed	-1.85	0.61	-3.03	-0.65
water source: spring-fed	-3.59	1.32	-6.10	-0.95
velocity	-0.99	2.72	-6.15	4.42

Table 3a Factors that explain variation in selenium concentration of Yuma Ridgway's rail head feathers collected from the Salton Sea, California, USA (2021). Phase 1 includes all models with $\Delta AIC_C \leq 2$, the null model, and the global model (sex*mass + age*mass). Phase 2 includes all models with $\Delta AIC_C \leq 2$, the informed null (top model from phase 1), and the global model (water source + velocity*marsh size). ΔAIC_C = the difference in Akaike Information Criterion for small sample sizes from the best fitting model; w_i = Akaike weight of the model. Potential explanatory variables include: age (juvenile vs adult); water source (spring-fed, river-fed, or ag-fed); velocity (two-week rolling average of the inflow velocity at the time of rail capture); marsh size (total hectares of continuous cattail marsh)

**Phase 1**		
**Model**	**ΔAIC_C**	**w_i**
null	0.00	0.78
global	2.58	0.22
**Phase 2**		
water source + marsh size	0.00	0.76
global	2.36	0.23
null	25.04	0.00

Table 3b Parameter estimates (Beta), standard errors (SE), and 95% confidence intervals for all predictor variables included in the top model (phase 2) to explain variation in selenium concentration of Yuma Ridgway's rail head feathers collected from the Salton Sea, California, USA (2021). water source (spring-fed, river-fed, or ag-fed [reference category]); velocity (two-week rolling average of the inflow velocity at the time of rail capture); marsh size (total hectares of continuous cattail marsh)

**Parameter**	**Beta**	**SE**	**Lower 95% CI**	**Upper 95% CI**
intercept	2.93	0.15	2.63	3.23
water source: river-fed	-0.86	0.18	-1.22	-0.51
water source: spring-fed	-1.50	0.28	-2.06	-0.93
marsh size	-0.001	0.0003	-0.001	-0.0004

Table 4 All selenium (Se; ppm dw) and biological data of Yuma Ridgway's rails sampled at the Salton Sea, California, USA (2020–2021). Rail ID plus a number (e.g., "_1") denotes capture and recapture events; BF = breast feathers; HF = head feathers; Inflow velocity = two-week rolling average of the marsh inflow velocity at the time of rail capture

Year	Rail ID	Blood Se	BF Se	HF Se	Sex	Age	Mass (g)	Water Source	Inflow Velocity (m^3/s)	Marsh Size (ha)
2020	R1	9.91	6.62	-	F	A	242	Ag	0.034	48
2020	R2	-	1.70	-	F	A	280	Ag	0.199	137
2020	R3	-	4.97	-	F	A	280	Ag	0.000	137
2020	R4	21.90	1.23	-	F	A	200	Ag	0.000	137
2020	R5	11.90	2.83	-	F	A	290	Ag	0.000	137
2020	R6	16.10	4.64	-	F	A	260	Ag	0.000	137
2020	R7	18.20	4.28	-	F	A	245	Ag	0.242	137
2020	R8	-	2.87	-	M	A	263	Ag	0.044	48
2020	R9	14.00	6.52	-	M	A	334	Ag	0.039	48
2020	R10	9.42	5.09	-	M	A	283	Ag	0.035	48
2020	R11	13.60	7.25	-	M	A	298	Ag	0.037	48
2020	R12	27.20	3.61	-	M	A	280	Ag	0.168	137
2020	R13	17.80	2.97	-	M	A	300	Ag	0.175	137
2020	R14	23.60	3.93	-	M	A	260	Ag	0.236	137
2020	R15	16.10	5.28	-	M	A	290	Ag	0.236	137
2020	R16	8.29	4.10	-	M	A	270	Ag	0.000	137
2020	R17	11.70	6.63	-	F	J	228	Ag	0.034	48
2020	R18	12.10	7.07	-	F	J	230	Ag	0.043	48
2020	R19	17.20	4.45	-	M	J	280	Ag	0.198	137
2020	R20	11.20	10.4	-	M	J	150	Ag	0.236	137
2020	R21	13.00	5.06	-	M	J	240	Ag	0.242	137
2020	R22	7.83	6.81	-	M	J	245	Ag	0.043	48
2021	R23	6.67	3.41	6.14	F	A	226	Ag	0.141	856
2021	R24	8.00	-	-	F	A	234	Ag	0.284	856
2021	R25_1	-	3.25	4.65	F	A	222	Ag	-	856
2021	R25_2	7.44	2.36	4.35	F	A	267	Ag	-	856
2021	R26	12.60	10.90	-	F	A	266	Ag	0.008	12
2021	R27	7.81	12.20	22.90	F	A	264	Ag	-	6
2021	R28	11.20	10.80	19.00	F	A	234	Ag	0.388	137
2021	R29	13.20	7.51	15.00	F	A	292	Ag	0.055	48
2021	R30	5.01	5.85	7.95	F	A	232	Ag	0.284	856
2021	R31	10.40	15.00	29.50	F	A	334	Ag	0.392	137
2021	R32	6.40	3.78	5.06	F	A	238	Ag	0.284	856
2021	R33	21.60	14.80	42.00	F	A	242	Ag	0.063	856
2021	R34	13.80	4.85	7.20	F	A	243	Ag	0.309	856
2021	R35	-	1.46	2.57	F	A	201	Ag	-	856
2021	R36	9.78	6.63	10.90	F	A	226	Ag	0.373	137
2021	R37	6.94	1.72	2.50	F	A	241	Ag	0.273	856

Year	Rail ID	Blood Se	BF Se	HF Se	Sex	Age	Mass (g)	Water Source	Inflow Velocity (m^3/s)	Marsh Size (ha)
2021	R38	5.65	5.25	2.15	F	A	245	Spring	-	66
2021	R39	6.59	6.66	5.40	F	A	266	Spring	-	66
2021	R40	18.00	8.47	38.00	F	A	231	Spring	-	66
2021	R41	5.05	1.37	2.00	F	A	276	Spring	-	30
2021	R42	5.13	2.46	2.50	M	A	276	Spring	-	66
2021	R43	5.01	1.57	1.84	M	A	302	Spring	-	30
2021	R44	5.30	-	-	M	J	-	Spring	-	66
2020	R45	5.61	1.59	-	F	A	211	River	0.028	11
2020	R46	4.11	1.70	-	F	A	214	River	0.028	11
2020	R47	7.36	6.02	-	F	A	243	River	0.087	42
2020	R48	5.56	2.12	-	F	A	249	River	0.085	42
2020	R49	2.49	1.10	-	F	A	221	River	0.084	42
2020	R50	9.52	2.47	-	F	A	240	River	0.084	42
2020	R51	24.80	8.18	-	M	A	268	River	0.028	11
2020	R52	8.15	2.88	-	M	A	289	River	0.028	11
2020	R53	11.30	8.07	-	M	A	284	River	0.024	4
2020	R54	5.90	3.84	-	M	A	290	River	0.028	11
2020	R55	7.20	4.76	-	M	A	279	River	0.087	42
2020	R56	6.96	5.19	-	M	A	279	River	0.085	42
2020	R57	2.30	1.32	-	M	A	253	River	0.084	42
2020	R58	9.52	4.52	-	M	A	278	River	0.084	42
2020	R59	4.68	1.94	-	M	A	281	River	0.084	42
2020	R60	7.80	3.64	-	F	J	225	River	0.085	42
2020	R61	9.29	8.69	-	F	J	208	River	0.084	42
2020	R62	7.96	4.27	-	M	J	325	River	0.085	42
2020	R63	8.57	9.85	-	M	J	269	River	0.085	42
2020	R64_1	1.02	-	-	M	J	192	River	0.084	42
2020	R65_1	7.28	6.81	-	M	J	303	River	0.084	42
2021	R66	6.16	7.41	13.50	F	A	303	River	0.062	42
2021	R67	6.36	6.57	8.84	F	A	295	River	0.038	4
2021	R68	6.47	2.74	3.40	F	A	254	River	0.039	11
2021	R69	27.80	5.45	11.10	F	A	205	River	0.056	42
2021	R70	8.43	5.59	5.20	F	A	275	River	0.056	67
2021	R71	13.10	12.80	14.70	F	A	236	River	-	67
2021	R72	8.29	2.84	4.12	F	A	329	River	0.056	191
2021	R73	10.10	2.96	3.34	F	A	217	River	0.028	67
2021	R74	5.00	5.21	5.71	F	A	237	River	0.169	191
2021	R65_2	10.10	3.96	3.91	M	A	307	River	0.062	42
2021	R75	10.30	4.65	9.64	M	A	308	River	0.062	42
2021	R76	5.66	13.70	22.00	M	A	309	River	0.062	42
2021	R77	7.63	5.37	5.66	M	A	310	River	0.038	4

Year	Rail ID	Blood Se	BF Se	HF Se	Sex	Age	Mass (g)	Water Source	Inflow Velocity (m^3/s)	Marsh Size (ha)
2021	R78	12.40	9.86	25.00	M	A	333	River	0.035	11
2021	R79	7.25	9.54	16.60	M	A	299	River	0.039	11
2021	R80	10.50	5.91	9.06	M	A	346	River	0.048	42
2021	R81	12.50	7.63	12.70	M	A	302	River	0.056	42
2021	R82	13.30	4.17	6.19	M	A	252	River	0.056	42
2021	R83	14.90	2.94	3.18	M	A	281	River	0.056	42
2021	R84	9.31	2.56	4.17	M	A	266	River	0.049	42
2021	R85	7.68	8.18	17.30	M	A	275	River	0.056	67
2021	R86	7.80	6.50	18.90	M	A	324	River	0.056	67
2021	R87	14.40	14.60	27.00	M	A	311	River	-	67
2021	R88	9.63	4.62	8.10	M	A	271	River	0.169	191
2021	R89	8.04	4.40	7.50	M	A	300	River	0.028	67
2021	R90	11.90	3.09	4.38	M	A	298	River	0.056	191
2021	R91	8.51	9.16	9.31	M	A	310	River	0.056	67
2021	R92	5.49	3.06	4.05	M	A	295	River	0.169	191
2021	R93	12.10	5.55	11.10	M	A	276	River	0.040	42
2021	R94	5.40	4.30	4.11	M	A	303	River	0.040	11
2021	R95	7.67	1.99	3.71	M	A	274	River	0.049	11
2021	R64_2	18.70	8.23	13.00	M	A	290	Ag	0.063	856
2021	R96	11.80	-	10.90	M	A	298	Ag	0.284	856
2021	R97	10.20	10.50	20.80	M	A	300	Ag	0.008	12
2021	R98	9.15	11.40	16.60	M	A	316	Ag	0.070	48
2021	R99	9.33	11.30	15.30	M	A	263	Ag	0.386	137
2021	R100	9.82	10.80	16.30	M	A	261	Ag	0.388	137
2021	R101	12.10	11.10	25.00	M	A	300	Ag	0.055	48
2021	R102	9.70	5.91	10.40	M	A	249	Ag	0.284	856
2021	R103	8.39	5.78	9.30	M	A	296	Ag	0.284	856
2021	R104_1	9.53	5.80	7.98	M	A	277	Ag	0.284	856
2021	R104_2	11.80	5.62	8.23	M	A	287	Ag	0.309	856
2021	R105	14.40	10.70	19.70	M	A	313	Ag	0.065	48
2021	R106	14.20	9.37	15.10	M	A	301	Ag	0.392	137
2021	R107	10.40	10.80	17.60	M	A	274	Ag	0.392	137
2021	R108	9.03	6.30	10.10	M	A	237	Ag	0.315	856
2021	R109	8.19	5.27	6.95	M	A	318	Ag	0.094	856
2021	R110	5.03	3.09	3.79	M	A	251	Ag	-	856
2021	R111	23.00	8.65	23.10	M	A	321	Ag	0.006	12
2021	R112	13.30	5.27	9.95	M	A	291	Ag	0.039	48
2021	R113	18.00	11.50	21.20	M	A	293	Ag	0.325	137
2021	R114	16.80	7.28	15.40	M	A	296	Ag	0.023	48
2021	R115	10.40	5.98	9.04	M	A	283	Ag	0.008	39
2021	R116	18.60	15.30	20.70	F	J	178	Ag	0.004	12

Year	Rail ID	Blood Se	BF Se	HF Se	Sex	Age	Mass (g)	Water Source	Inflow Velocity (m^3/s)	Marsh Size (ha)
2021	R117	8.02	12.70	24.70	F	J	204	Ag	0.004	12
2021	R118	9.64	3.77	8.92	M	J	272	Ag	0.243	856
2021	R119	12.40	11.60	16.30	M	J	260	Ag	0.266	137

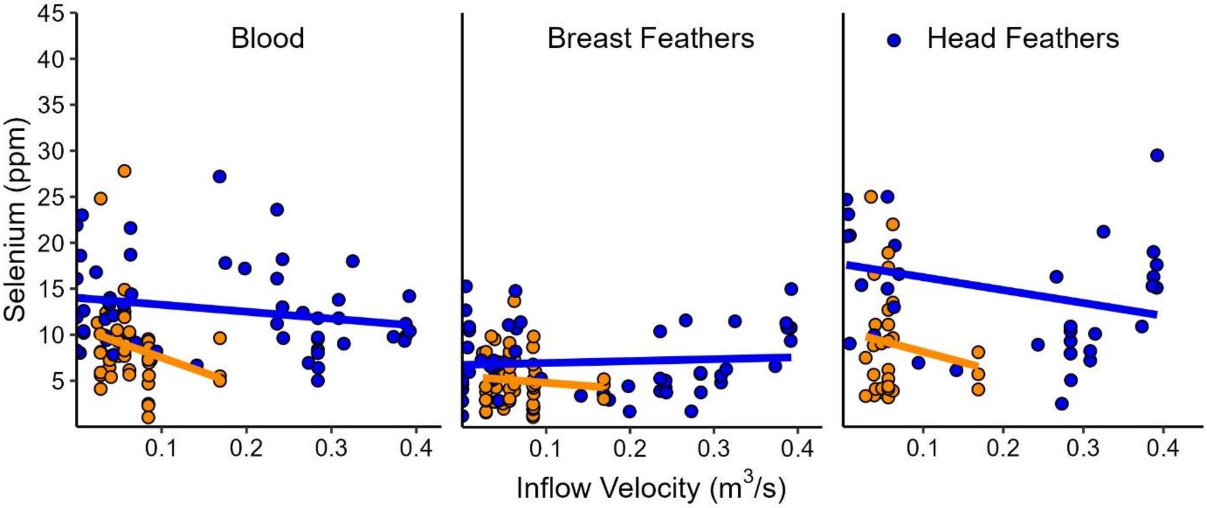

Fig. 1 Relationship of Yuma Ridgway's rail blood, breast feather, and head feather (2021 only) selenium concentration (ppm dw) to the inflow velocity (two-week rolling average at rail capture date) in river-fed (orange) and ag-fed (blue) marshes at the Salton Sea, California, USA (2020–2021)
