# Supplementary material for: Irrigated agriculture influences selenium levels in an endangered marsh bird
Source: Environ Monit Assess. 2025 Sep 24;197(10):1142. doi: 10.1007/s10661-025-14533-1 (PMC12460551; doi:10.1007/s10661-025-14533-1)
Supplement: Supplementary file 2 — Online Resource 2 (PDF 195 MB) [file 10661_2025_14533_MOESM2_ESM.pdf]

Online Resource 2

Irrigated agriculture influences selenium levels in an endangered marsh bird

Environmental Monitoring and Assessment

Cydney M. Yost, Kathryn M. Sliwa, Razia Shafique-Sabir, Jonathan Shore, & Courtney J. Conway

Cydney M. Yost (corresponding author), Idaho Cooperative Fish & Wildlife Research Unit, University of Idaho, Department of Fish & Wildlife Sciences, Moscow, ID, USA, e-mail: cydney.yost@gmail.com

Supplementary tables and raw data related to Yuma Ridgway's rail eggshells and egg content

Table 1a Factors that explain variation in selenium concentration of Yuma Ridgway's rail egg content collected from the Salton Sea, California, USA (2021–2022). The table includes all models with $\Delta AIC_C \leq 2$, the null model, and the global model (water source + velocity*marsh size + distance to inflow). ΔAIC_C = the difference in Akaike Information Criterion for small sample sizes from the best fitting model; w_i = Akaike weight of the model. Potential explanatory variables include: water source (river-fed or ag-fed); velocity (two-week rolling average of the inflow velocity at the nest end date); marsh size (total hectares of continuous cattail marsh); distance to inflow (distance of nest to marsh inflow)

**Model**	**ΔAIC_C**	**w_i**
velocity	0.00	0.50
water source + velocity	1.68	0.21
null	2.71	0.13
global	26.54	0.00

Table 1b Parameter estimate (Beta), standard error (SE), and 95% confidence interval for the top model to explain variation in selenium concentration of Yuma Ridgway's rail egg contents collected from the Salton Sea, California, USA (2021–2022)

**Parameter**	**Beta**	**SE**	**Lower 95% CI**	**Upper 95% CI**
intercept	4.38	1.65	0.51	7.95
velocity	-8.13	7.45	-23.79	7.16

Table 2a Factors that explain variation in selenium concentration of Yuma Ridgway's rail eggshells collected from the Salton Sea, California, USA (2020–2022). The table includes all models with $\Delta AIC_C \leq 2$, the null model, and the global model (water source + velocity*marsh size + distance to inflow). ΔAIC_C = the difference in Akaike Information Criterion for small sample sizes from the best fitting model; w_i = Akaike weight of the model. Potential explanatory variables include: water source (spring-fed, river-fed, or ag-fed); velocity (two-week rolling average of the inflow velocity at the nest end date); marsh size (total hectares of continuous cattail marsh); distance to inflow (distance of nest to marsh inflow)

**Model**	**ΔAIC_C**	**w_i**
null	0.00	0.52
velocity	0.48	0.41
global	31.09	0.00

Table 2b Parameter estimate (Beta), standard error (SE), and 95% confidence interval for the top model to explain variation in selenium concentration of Yuma Ridgway's rail eggshells collected from the Salton Sea, California, USA (2020–2022)

**Parameter**	**Beta**	**SE**	**Lower 95% CI**	**Upper 95% CI**
intercept	0.65	0.15	0.28	0.96

Table 3 All selenium (Se; ppm dw) data of Yuma Ridgway's rail eggs sampled from marshes of three different water sources (spring-fed, river-fed, ag-fed) at the Salton Sea, California, USA (2020–2022). Nest ID = Parent rail ID plus a number (e.g., "_1") denoting eggs from same parent but different nests; Inflow velocity = two-week rolling average of the marsh inflow velocity at nest end date; Parent rails were unknown (UNK) for incidentally found nests

Year	Nest ID	Egg Content Se	Eggshell Se	Water Source	Marsh Size (ha)	Inflow Velocity (m^3/s)	Distance to Inflow (m)
2020	R11	-	0.58	Ag	48	0.042	504.44
2020	UNK	-	0.22	Ag	137	0.242	269.32
2021	R26_1	7.11	1.23	Ag	12	-	-
2021	R26_2	2.84	0.43	Ag	12	0.006	108.16
2021	R98_1	5.75	0.64	Ag	48	0.030	769.20
2021	R98_2	5.27	1.11	Ag	48	0.030	767.34
2021	R98_2	-	0.28	Ag	48	0.030	767.34
2021	R99_1	-	0.65	Ag	137	0.258	615.06
2021	R99_2	-	0.53	Ag	137	0.256	580.08
2021	R100	-	0.94	Ag	137	0.395	172.97
2021	R28_1	-	0.51	Ag	137	0.400	461.40
2021	R28_1	-	2.24	Ag	137	0.400	461.40
2021	R29	-	0.48	Ag	48	0.050	676.62
2021	R30_1	3.09	-	Ag	856	0.277	98.56
2021	R30_1	3.86	-	Ag	856	0.277	98.56
2021	R30_1	-	0.47	Ag	856	0.277	98.56
2021	R31	-	0.61	Ag	137	0.397	722.29
2021	R32	-	0.34	Ag	856	0.222	888.85
2021	R105_1	7.50	0.41	Ag	48	0.032	502.68
2021	R105_1	8.34	0.97	Ag	48	0.032	502.68
2021	R105_1	-	0.62	Ag	48	0.032	502.68
2021	R1	-	0.89	Ag	48	0.030	762.39
2021	R64	-	2.39	Ag	856	0.063	299.16
2021	UNK	-	0.58	Ag	137	0.400	329.82
2021	UNK	-	2.26	Ag	137	0.400	456.08
2021	UNK	-	2.76	Ag	856	0.068	361.57
2022	R117	-	0.29	Ag	856	0.283	1011.21
2022	R120	-	0.31	Ag	856	0.228	352.72
2022	UNK	-	0.26	Ag	48	0.073	680.09
2021	R43	-	0.25	Spring	30	-	314.08
2022	R121_1	-	0.68	Spring	66	-	571.55
2022	R121_2	-	0.78	Spring	66	-	565.90
2020	R53	-	0.45	River	4	0.018	116.91
2020	UNK	-	0.45	River	42	0.084	341.63
2021	R66_1	1.99	1.44	River	42	-	-
2021	R66_2	2.04	0.18	River	42	0.051	437.00
2021	R66_2	2.60	0.22	River	42	0.051	437.00

Year	Nest ID	Egg Content Se	Eggshell Se	Water Source	Marsh Size (ha)	Inflow Velocity (m^3/s)	Distance to Inflow (m)
2021	R66_2	-	0.17	River	42	0.051	437.00
2021	R77_1	-	0.38	River	4	0.033	201.18
2021	R77_2	-	0.39	River	4	0.047	181.72
2021	R78_1	9.10	2.67	River	11	0.043	60.09
2021	R78_1	-	0.72	River	11	0.043	60.09
2021	R68	-	0.70	River	11	0.051	267.65
2021	R85_1	-	0.42	River	67	0.056	895.81
2022	R85_2	-	0.40	River	67	0.056	849.56
2022	R85_3	1.69	0.60	River	67	0.087	905.66
2022	R85_3	2.05	0.45	River	67	0.087	905.66
2022	R85_3	2.07	0.59	River	67	0.087	905.66
2022	R85_3	-	0.44	River	67	0.087	905.66
2022	R82	-	0.34	River	42	0.067	581.64
2022	R122	-	0.71	River	4	0.016	89.95
2022	R123	-	0.70	River	11	-	95.28
