# Supplementary material for: Irrigated agriculture influences selenium levels in an endangered marsh bird
Source: Environ Monit Assess. 2025 Sep 24;197(10):1142. doi: 10.1007/s10661-025-14533-1 (PMC12460551; doi:10.1007/s10661-025-14533-1)
Supplement: Supplementary file 3 — Online Resource 3 (PDF 610 KB) [file 10661_2025_14533_MOESM3_ESM.pdf]

Online Resource 3

Irrigated agriculture influences selenium levels in an endangered marsh bird

Environmental Monitoring and Assessment

Cydney M. Yost, Kathryn M. Sliwa, Razia Shafique-Sabir, Jonathan Shore, & Courtney J. Conway

Cydney M. Yost (corresponding author), Idaho Cooperative Fish & Wildlife Research Unit, University of Idaho, Department of Fish & Wildlife Sciences, Moscow, ID, USA, e-mail: cydney.yost@gmail.com

Supplementary tables, figures, and raw data related to Yuma Ridgway's rail prey

Table 1 The total number of prey (Total) and percent composition (% Comp) of prey caught in traps in spring-fed, river-fed, and ag-fed marshes at the Salton Sea, California, USA (2022–2023). BG = bluegill sunfish (*Lepomis macrochirus*); WM = western mosquitofish (*Gambusia affinis*); SM = sailfin molly (*Poecilia latipinna*); CC = common carp (*Cyprinus carpio*); OF = other fish (*Cyprinella*, *Micropterus*, *Morone* spp); MT = Mozambique tilapia (*Oreochromis mossambicus*); IN = Belostomatidae, Corixidae, and *Coleoptera* sp; CF = red swamp crayfish (*Procambarus clarkii*); SH = shrimp (Palaemonidae sp); BT = American bullfrog tadpoles (*Lithobates catesbeianus*)

	**Water Source**					
	**Spring**		**River**		**Ag**	
**Prey Type**	Total	% Comp	Total	% Comp	Total	% Comp
BG	-	-	223	4.9	-	-
WM	210	71.2	1535	33.6	502	27.3
SM	30	10.2	72	1.6	294	16
CC	-	-	46	1	-	0
OF	-	-	54	1.2	4	0.2
MT	-	-	-	-	18	1
IN	7	2.4	171	3.7	79	4.3
CF	12	4.1	1318	28.9	428	23.3
SH	12	4.1	845	18.5	468	25.4
BT	24	8.1	301	6.6	46	2.5

Table 2a Factors that explain variation in selenium concentration of mosquitofish (Yuma Ridgway's rail prey) collected from the Salton Sea, California, USA (2020–2022). The table includes all models with $\Delta AIC_C \leq 2$, the null model, and the global model (water source + velocity*marsh size + distance to inflow). ΔAIC_C = the difference in Akaike Information Criterion for small sample sizes from the best fitting model; w_i = Akaike weight of the model. Potential explanatory variables include: water source (spring-fed, river-fed, or ag-fed); velocity (two-week rolling average of the inflow velocity at the time of prey capture); marsh size (total hectares of continuous cattail marsh); distance to inflow (distance of sampling location to marsh inflow)

**Model**	**ΔAIC_C**	**w_i**
water source + velocity	0.00	0.70
water source + velocity + marsh size + velocity*marsh size	1.78	0.29
global	12.63	0.00
null	31.71	0.00

Table 2b Parameter estimates (Beta), standard errors (SE), and 95% confidence intervals for all predictor variables included in the top model to explain variation in selenium concentration of mosquitofish (Yuma Ridgway's rail prey) collected from the Salton Sea, California, USA (2020–2022). Water source (spring-fed, river-fed, or ag-fed [reference category]); velocity (two-week rolling average of inflow velocity at the time of prey capture)

**Parameter**	**Beta**	**SE**	**Lower 95% CI**	**Upper 95% CI**
intercept	8.02	0.61	6.85	9.17
water source: river-fed	-3.05	0.54	-4.10	-2.00
water source: spring-fed	-3.45	0.80	-4.99	-1.82
velocity	-9.33	2.99	-15.07	-3.35

Table 3a Factors that explain variation in selenium concentration of crayfish (Yuma Ridgway's rail prey) collected from the Salton Sea, California, USA (2020–2022). The table includes all models with $\Delta AIC_C \leq 2$, the null model, and the global model (water source + velocity*marsh size + distance to inflow). ΔAIC_C = the difference in Akaike Information Criterion for small sample sizes from the best fitting model; w_i = Akaike weight of the model. Potential explanatory variables include: water source (spring-fed, river-fed, or ag-fed); velocity (two-week rolling average of the inflow velocity at the time of prey capture); marsh size (total hectares of continuous cattail marsh); distance to inflow (distance of prey sampling location to marsh inflow)

**Model**	**ΔAIC_C**	**w_i**
water source + velocity	0.00	0.94
null	21.04	0.00
global	27.83	0.00

Table 3b Parameter estimates (Beta), standard errors (SE), and 95% confidence intervals for all predictor variables included in the top model to explain variation in selenium concentration of crayfish (Yuma Ridgway's rail prey) collected from the Salton Sea, California, USA (2020–2022). Water source (spring-fed, river-fed, or ag-fed [reference category]); velocity (two-week rolling average of the inflow velocity at the time of prey capture)

**Parameter**	**Beta**	**SE**	**Lower 95% CI**	**Upper 95% CI**
intercept	4.97	0.35	4.28	5.58
water source: river-fed	-1.58	0.31	-2.11	-0.90
water source: spring-fed	-1.18	0.59	-2.38	-0.08
velocity	-6.35	2.28	-10.76	-1.83

Table A4 All selenium (Se; ppm dw) concentration data from Yuma Ridgway's rail prey in marshes of three different water sources (spring-fed, river-fed, and ag-fed) at the Salton Sea, California, USA (2020–2022). Inflow velocity = two-week rolling average of the inflow velocity at the time of prey capture; marsh size = total hectares of continuous cattail marsh; distance to inflow = distance of prey sampling location to marsh inflow; WM = western mosquitofish (*Gambusia affinis*); CF = red swamp crayfish (*Procambarus clarkii*); SM = sailfin molly (*Poecilia latipinna*); CC = common carp (*Cyprinus carpio*); BG = bluegill sunfish (*Lepomis macrochirus*); MT = Mozambique tilapia (*Oreochromis mossambicus*); OF = other fish (*Cyprinella*, *Micropterus*, *Morone* spp); SH = shrimp (*Palaemonidae* sp); BT = American bullfrog tadpoles (*Lithobates catesbeianus*); IN = Belostomatidae, Corixidae and *Coleoptera* sp

Year	Prey Type	Se	Water Source	Inflow Velocity (m^3/s)	Marsh Size (ha)	Distance to Inflow (m)
2021	WM	1.40	Spring	-	30	273.13
2021	WM	0.47	Spring	-	30	281.90
2021	WM	1.04	Spring	-	30	296.27
2021	WM	1.02	Spring	-	30	338.07
2021	WM	0.83	Spring	-	30	338.67
2021	WM	1.37	Spring	-	30	355.39
2021	WM	5.73	Spring	-	66	53.76
2021	WM	6.55	Spring	-	66	91.17
2021	WM	6.40	Spring	-	66	145.59
2021	WM	8.29	Spring	-	66	149.02
2021	WM	9.71	Spring	-	66	171.00
2021	WM	3.25	Spring	-	66	184.61
2021	WM	11.20	Spring	-	66	207.66
2021	WM	6.10	Spring	-	66	234.01
2021	WM	2.34	Spring	-	66	244.24
2021	WM	3.67	Spring	-	66	260.46
2021	WM	5.12	Spring	-	66	568.88
2021	WM	4.41	Spring	-	66	569.21
2021	WM	3.80	Spring	-	66	574.38
2021	WM	4.26	Spring	-	66	652.73
2021	WM	4.17	Spring	-	66	711.13
2021	WM	3.93	Spring	-	66	730.24
2022	WM	3.63	Spring	-	66	131.00
2022	WM	3.50	Spring	-	66	135.32

Year	Prey Type	Se	Water Source	Inflow Velocity (m^3/s)	Marsh Size (ha)	Distance to Inflow (m)
2022	WM	8.11	Spring	-	66	209.53
2022	WM	9.19	Spring	-	66	209.59
2022	WM	9.89	Spring	-	66	212.12
2020	WM	9.63	River	0.028	11	53.23
2020	WM	13.40	River	0.028	11	59.91
2020	WM	15.60	River	0.028	11	65.17
2020	WM	1.35	River	0.028	11	66.32
2020	WM	1.81	River	0.028	11	282.59
2020	WM	1.57	River	0.028	11	309.54
2020	WM	3.82	River	0.084	42	10.96
2020	WM	3.04	River	0.084	42	111.75
2020	WM	4.54	River	0.084	42	121.37
2020	WM	4.08	River	0.084	42	164.47
2020	WM	4.29	River	0.084	42	164.93
2020	WM	1.50	River	0.084	42	254.55
2020	WM	1.51	River	0.084	42	315.24
2020	WM	3.30	River	0.084	42	324.19
2020	WM	3.77	River	0.084	42	332.00
2020	WM	2.54	River	0.084	42	365.93
2020	WM	0.52	River	0.084	42	403.62
2020	WM	1.72	River	0.084	42	487.64
2020	WM	2.01	River	0.084	42	533.29
2020	WM	1.68	River	0.084	42	765.15
2020	WM	4.12	River	0.084	42	773.12
2020	WM	5.86	River	0.085	42	16.51
2021	WM	2.93	River	0.020	4	201.19
2021	WM	3.19	River	0.024	4	210.28
2021	WM	3.60	River	0.044	11	218.92
2021	WM	2.22	River	0.044	11	317.98
2021	WM	1.62	River	0.051	11	422.44

Year	Prey Type	Se	Water Source	Inflow Velocity (m^3/s)	Marsh Size (ha)	Distance to Inflow (m)
2021	WM	2.33	River	0.053	11	185.07
2021	WM	6.35	River	0.053	11	272.73
2021	WM	1.39	River	0.053	11	278.35
2021	WM	2.31	River	0.053	11	425.40
2021	WM	3.33	River	0.054	11	192.40
2021	WM	1.95	River	0.057	11	235.39
2021	WM	2.22	River	0.057	11	341.75
2021	WM	2.19	River	0.057	11	386.16
2021	WM	4.81	River	0.040	42	105.93
2021	WM	2.06	River	0.040	42	275.19
2021	WM	2.02	River	0.040	42	324.27
2021	WM	14.90	River	0.040	42	585.15
2021	WM	15.30	River	0.040	42	607.56
2021	WM	14.70	River	0.040	42	650.31
2021	WM	5.34	River	0.042	42	121.52
2021	WM	5.56	River	0.042	42	148.20
2021	WM	3.92	River	0.042	42	152.10
2021	WM	5.65	River	0.042	42	174.69
2021	WM	3.95	River	0.042	42	196.72
2021	WM	1.56	River	0.042	42	380.89
2021	WM	5.70	River	0.044	42	124.01
2021	WM	4.26	River	0.044	42	148.87
2021	WM	4.41	River	0.046	42	172.72
2021	WM	5.62	River	0.028	67	213.11
2021	WM	5.55	River	0.028	67	258.03
2021	WM	5.55	River	0.028	67	258.03
2021	WM	5.62	River	0.028	67	365.27
2021	WM	3.53	River	0.028	67	391.83
2021	WM	4.76	River	0.028	67	408.45
2021	WM	3.08	River	0.028	67	941.44

Year	Prey Type	Se	Water Source	Inflow Velocity (m^3/s)	Marsh Size (ha)	Distance to Inflow (m)
2021	WM	3.15	River	0.028	67	954.22
2021	WM	3.12	River	0.028	67	968.88
2021	WM	4.93	River	0.056	67	725.29
2021	WM	5.55	River	-	67	413.47
2021	WM	4.81	River	-	67	663.02
2021	WM	4.28	River	-	67	694.33
2021	WM	8.63	River	0.056	191	26.56
2021	WM	6.52	River	0.056	191	36.20
2021	WM	8.26	River	0.056	191	73.53
2021	WM	6.21	River	0.169	191	236.03
2021	WM	4.25	River	0.169	191	253.91
2021	WM	14.50	River	0.169	191	280.11
2021	WM	11.40	River	0.169	191	291.20
2021	WM	3.96	River	0.169	191	296.96
2021	WM	6.04	River	0.169	-	-
2021	WM	6.09	River	0.169	-	-
2021	WM	3.81	River	-	-	-
2021	WM	4.04	River	-	-	-
2021	WM	4.55	River	-	-	-
2021	WM	4.55	River	-	-	-
2022	WM	3.93	River	0.029	4	292.05
2022	WM	4.10	River	-	11	272.35
2022	WM	4.63	River	-	11	272.69
2022	WM	6.22	River	0.014	42	5.92
2022	WM	4.46	River	0.014	42	8.13
2022	WM	8.28	River	0.014	42	12.18
2022	WM	3.99	River	0.014	42	13.60
2022	WM	2.05	River	0.014	42	15.07
2022	WM	2.07	River	0.014	42	722.70
2022	WM	2.41	River	0.014	42	727.01

Year	Prey Type	Se	Water Source	Inflow Velocity (m^3/s)	Marsh Size (ha)	Distance to Inflow (m)
2022	WM	1.85	River	0.014	42	731.80
2022	WM	2.12	River	0.014	42	740.32
2022	WM	1.92	River	0.014	42	740.58
2022	WM	5.01	River	0.056	42	76.77
2022	WM	4.69	River	0.058	42	74.83
2022	WM	4.01	River	0.058	42	94.14
2022	WM	2.90	River	0.058	42	107.30
2022	WM	2.44	River	0.056	67	941.74
2022	WM	2.44	River	0.057	67	941.81
2022	WM	2.52	River	0.057	67	951.03
2020	WM	8.37	Ag	0.043	48	440.29
2020	WM	11.90	Ag	0.043	48	614.41
2020	WM	5.41	Ag	0.043	48	833.57
2020	WM	4.63	Ag	0.043	48	857.09
2020	WM	7.80	Ag	0.046	48	457.14
2020	WM	6.23	Ag	0.046	48	496.56
2020	WM	7.60	Ag	0.046	48	736.39
2020	WM	5.33	Ag	0.200	137	167.85
2020	WM	7.14	Ag	0.210	137	126.58
2020	WM	6.06	Ag	0.210	137	487.85
2020	WM	5.96	Ag	0.236	137	322.97
2020	WM	6.27	Ag	0.236	137	363.00
2020	WM	4.53	Ag	0.242	137	129.16
2020	WM	5.90	Ag	0.242	137	338.73
2020	WM	12.20	Ag	0.242	137	919.81
2020	WM	4.11	Ag	0.242	137	991.71
2020	WM	9.25	Ag	-	137	77.92
2021	WM	5.49	Ag	-	6	11.74
2021	WM	8.03	Ag	-	6	172.63
2021	WM	6.94	Ag	-	6	198.07

Year	Prey Type	Se	Water Source	Inflow Velocity (m^3/s)	Marsh Size (ha)	Distance to Inflow (m)
2021	WM	9.18	Ag	0.004	12	66.51
2021	WM	6.75	Ag	0.008	12	22.41
2021	WM	6.45	Ag	0.008	12	40.90
2021	WM	10.50	Ag	0.008	12	54.87
2021	WM	11.60	Ag	0.008	12	66.51
2021	WM	12.20	Ag	0.008	12	80.07
2021	WM	5.45	Ag	0.008	12	80.86
2021	WM	6.56	Ag	0.024	48	872.44
2021	WM	6.03	Ag	0.024	48	882.54
2021	WM	6.34	Ag	0.024	48	890.78
2021	WM	7.30	Ag	0.031	48	678.31
2021	WM	4.97	Ag	0.032	48	1003.62
2021	WM	10.10	Ag	0.032	48	644.97
2021	WM	7.80	Ag	0.033	48	541.86
2021	WM	4.59	Ag	0.033	48	1012.32
2021	WM	3.71	Ag	0.033	48	1057.35
2021	WM	9.24	Ag	0.036	48	536.38
2021	WM	6.94	Ag	0.040	48	661.99
2021	WM	10.30	Ag	0.040	48	680.06
2021	WM	7.21	Ag	0.226	137	381.04
2021	WM	7.38	Ag	0.226	137	410.89
2021	WM	8.54	Ag	0.226	137	446.31
2021	WM	10.80	Ag	0.226	137	725.37
2021	WM	9.40	Ag	0.226	137	742.33
2021	WM	8.40	Ag	0.226	137	752.26
2021	WM	4.82	Ag	0.250	137	8.31
2021	WM	6.77	Ag	0.250	137	25.60
2021	WM	5.00	Ag	0.250	137	37.79
2021	WM	5.85	Ag	0.250	137	72.57
2021	WM	5.69	Ag	0.250	137	84.16

Year	Prey Type	Se	Water Source	Inflow Velocity (m^3/s)	Marsh Size (ha)	Distance to Inflow (m)
2021	WM	5.73	Ag	0.250	137	92.70
2021	WM	5.35	Ag	0.266	137	501.49
2021	WM	7.07	Ag	0.266	137	548.90
2021	WM	7.74	Ag	0.266	137	605.42
2021	WM	5.58	Ag	0.091	856	13.53
2021	WM	12.60	Ag	0.091	856	317.14
2021	WM	8.47	Ag	0.092	856	28.54
2021	WM	7.79	Ag	0.092	856	40.18
2021	WM	16.30	Ag	0.092	856	280.73
2021	WM	14.20	Ag	0.092	856	347.82
2021	WM	3.45	Ag	0.137	856	108.62
2021	WM	3.62	Ag	0.141	856	73.78
2021	WM	15.40	Ag	0.155	856	264.63
2021	WM	11.40	Ag	0.155	856	362.82
2021	WM	4.27	Ag	0.238	856	59.13
2021	WM	4.34	Ag	0.238	856	89.24
2021	WM	3.54	Ag	0.252	856	112.23
2021	WM	5.33	Ag	0.261	856	85.65
2021	WM	4.29	Ag	0.261	856	120.24
2021	WM	4.74	Ag	0.263	856	133.40
2021	WM	2.00	Ag	0.312	856	1027.39
2021	WM	1.84	Ag	0.313	856	1001.60
2021	WM	1.94	Ag	0.313	856	1021.51
2022	WM	5.17	Ag	0.012	12	24.50
2022	WM	7.77	Ag	0.012	12	79.41
2022	WM	8.45	Ag	0.012	12	85.45
2022	WM	6.35	Ag	0.029	12	1.68
2022	WM	7.71	Ag	0.029	12	4.72
2022	WM	8.73	Ag	0.029	12	87.10
2022	WM	7.22	Ag	0.068	48	655.36

Year	Prey Type	Se	Water Source	Inflow Velocity (m^3/s)	Marsh Size (ha)	Distance to Inflow (m)
2022	WM	3.45	Ag	0.068	48	664.70
2022	WM	6.95	Ag	0.068	48	671.43
2022	WM	8.59	Ag	0.070	48	458.50
2022	WM	6.43	Ag	0.070	48	474.69
2022	WM	7.61	Ag	0.070	48	483.05
2022	WM	5.41	Ag	0.194	856	1510.95
2022	WM	4.12	Ag	0.194	856	1527.16
2022	WM	3.64	Ag	0.260	856	123.01
2022	WM	5.85	Ag	0.260	856	127.28
2022	WM	1.85	Ag	0.266	856	914.42
2022	WM	1.85	Ag	0.266	856	931.88
2022	WM	3.99	Ag	0.278	856	71.92
2022	WM	4.38	Ag	0.278	856	99.86
2021	CF	2.19	Spring	-	66	53.76
2021	CF	4.73	Spring	-	66	91.17
2021	CF	4.07	Spring	-	66	145.59
2021	CF	3.70	Spring	-	66	149.02
2021	CF	5.86	Spring	-	66	207.66
2021	CF	2.32	Spring	-	66	244.24
2021	CF	3.25	Spring	-	66	568.88
2021	CF	2.15	Spring	-	66	569.21
2021	CF	2.88	Spring	-	66	574.38
2021	CF	2.55	Spring	-	66	711.13
2021	CF	2.44	Spring	-	66	730.24
2021	CF	2.69	Spring	-	66	731.37
2022	CF	3.68	Spring	-	66	135.32
2022	CF	4.94	Spring	-	66	209.53
2022	CF	5.73	Spring	-	66	209.59
2022	CF	6.07	Spring	-	66	212.12
2020	CF	4.04	River	0.028	11	53.23

Year	Prey Type	Se	Water Source	Inflow Velocity (m^3/s)	Marsh Size (ha)	Distance to Inflow (m)
2020	CF	5.73	River	0.028	11	53.23
2020	CF	6.17	River	0.028	11	53.23
2020	CF	8.07	River	0.028	11	53.23
2020	CF	11.90	River	0.028	11	53.23
2020	CF	4.02	River	0.028	11	59.91
2020	CF	5.36	River	0.028	11	59.91
2020	CF	5.94	River	0.028	11	59.91
2020	CF	8.16	River	0.028	11	59.91
2020	CF	9.71	River	0.028	11	59.91
2020	CF	6.94	River	0.028	11	65.17
2020	CF	8.18	River	0.028	11	65.17
2020	CF	8.95	River	0.028	11	65.17
2020	CF	12.20	River	0.028	11	65.17
2020	CF	12.50	River	0.028	11	65.17
2020	CF	1.05	River	0.028	11	66.32
2020	CF	1.16	River	0.028	11	66.32
2020	CF	1.32	River	0.028	11	66.32
2020	CF	1.34	River	0.028	11	66.32
2020	CF	1.51	River	0.028	11	66.32
2020	CF	2.04	River	0.028	11	212.18
2020	CF	2.07	River	0.028	11	212.18
2020	CF	1.68	River	0.028	11	219.03
2020	CF	1.92	River	0.028	11	219.03
2020	CF	2.18	River	0.028	11	219.03
2020	CF	2.34	River	0.028	11	219.03
2020	CF	2.57	River	0.028	11	219.03
2020	CF	1.42	River	0.028	11	282.59
2020	CF	1.45	River	0.028	11	282.59
2020	CF	1.51	River	0.028	11	282.59
2020	CF	1.84	River	0.028	11	282.59

Year	Prey Type	Se	Water Source	Inflow Velocity (m^3/s)	Marsh Size (ha)	Distance to Inflow (m)
2020	CF	2.78	River	0.028	11	282.59
2020	CF	1.16	River	0.028	11	309.54
2020	CF	1.18	River	0.028	11	309.54
2020	CF	1.39	River	0.028	11	309.54
2020	CF	3.29	River	0.084	42	10.96
2020	CF	3.35	River	0.084	42	10.96
2020	CF	3.54	River	0.084	42	10.96
2020	CF	4.21	River	0.084	42	10.96
2020	CF	4.35	River	0.084	42	10.96
2020	CF	2.86	River	0.084	42	111.75
2020	CF	3.05	River	0.084	42	111.75
2020	CF	3.46	River	0.084	42	111.75
2020	CF	3.91	River	0.084	42	111.75
2020	CF	4.88	River	0.084	42	111.75
2020	CF	2.70	River	0.084	42	164.47
2020	CF	2.75	River	0.084	42	164.47
2020	CF	3.04	River	0.084	42	164.47
2020	CF	2.67	River	0.084	42	164.93
2020	CF	2.94	River	0.084	42	164.93
2020	CF	2.95	River	0.084	42	164.93
2020	CF	3.30	River	0.084	42	164.93
2020	CF	3.35	River	0.084	42	164.93
2020	CF	0.92	River	0.084	42	254.55
2020	CF	0.99	River	0.084	42	254.55
2020	CF	1.00	River	0.084	42	254.55
2020	CF	1.16	River	0.084	42	254.55
2020	CF	1.86	River	0.084	42	254.55
2020	CF	1.57	River	0.084	42	279.92
2020	CF	2.32	River	0.084	42	324.19
2020	CF	2.37	River	0.084	42	324.19

Year	Prey Type	Se	Water Source	Inflow Velocity (m^3/s)	Marsh Size (ha)	Distance to Inflow (m)
2020	CF	2.65	River	0.084	42	324.19
2020	CF	3.23	River	0.084	42	324.19
2020	CF	2.04	River	0.084	42	332.00
2020	CF	3.32	River	0.084	42	332.00
2020	CF	1.30	River	0.084	42	365.93
2020	CF	2.00	River	0.084	42	365.93
2020	CF	2.06	River	0.084	42	365.93
2020	CF	2.63	River	0.084	42	365.93
2020	CF	2.78	River	0.084	42	365.93
2020	CF	0.64	River	0.084	42	765.15
2020	CF	0.84	River	0.084	42	765.15
2020	CF	1.35	River	0.084	42	765.15
2020	CF	1.47	River	0.084	42	773.12
2020	CF	1.50	River	0.084	42	773.12
2020	CF	1.51	River	0.084	42	773.12
2020	CF	1.53	River	0.084	42	773.12
2020	CF	1.79	River	0.084	42	773.12
2021	CF	2.50	River	0.020	4	201.19
2021	CF	2.59	River	0.024	4	189.20
2021	CF	2.84	River	0.024	4	210.28
2021	CF	2.28	River	0.043	11	311.99
2021	CF	4.98	River	0.044	11	69.36
2021	CF	1.92	River	0.044	11	218.92
2021	CF	1.60	River	0.044	11	317.98
2021	CF	1.27	River	0.052	11	460.00
2021	CF	2.04	River	0.053	11	185.07
2021	CF	2.78	River	0.053	11	272.73
2021	CF	0.94	River	0.053	11	278.35
2021	CF	1.26	River	0.053	11	288.75
2021	CF	2.07	River	0.053	11	425.40

Year	Prey Type	Se	Water Source	Inflow Velocity (m^3/s)	Marsh Size (ha)	Distance to Inflow (m)
2021	CF	2.70	River	0.054	11	178.74
2021	CF	2.41	River	0.054	11	192.40
2021	CF	1.06	River	0.057	11	235.39
2021	CF	2.19	River	0.057	11	341.75
2021	CF	2.26	River	0.057	11	386.16
2021	CF	3.02	River	0.040	42	275.19
2021	CF	3.76	River	0.042	42	121.52
2021	CF	2.64	River	0.042	42	148.20
2021	CF	2.99	River	0.042	42	152.10
2021	CF	0.90	River	0.042	42	380.89
2021	CF	2.98	River	0.042	42	460.00
2021	CF	4.32	River	0.044	42	124.01
2021	CF	3.26	River	0.044	42	148.87
2021	CF	2.37	River	0.046	42	172.72
2021	CF	2.92	River	0.028	67	365.27
2021	CF	1.79	River	0.028	67	941.44
2021	CF	1.58	River	0.028	67	968.88
2021	CF	2.75	River	-	67	413.47
2021	CF	2.92	River	-	67	663.02
2021	CF	2.31	River	-	67	694.33
2021	CF	2.86	River	0.169	191	253.91
2022	CF	3.88	River	0.006	4	61.63
2022	CF	6.99	River	0.006	4	82.13
2022	CF	7.74	River	0.006	4	95.78
2022	CF	3.71	River	0.029	4	11.49
2022	CF	5.97	River	0.029	4	290.27
2022	CF	3.19	River	0.029	4	292.63
2022	CF	5.14	River	-	11	7.87
2022	CF	4.02	River	-	11	71.15
2022	CF	2.31	River	-	11	191.47

Year	Prey Type	Se	Water Source	Inflow Velocity (m^3/s)	Marsh Size (ha)	Distance to Inflow (m)
2022	CF	5.42	River	-	11	220.17
2022	CF	4.03	River	-	11	263.74
2022	CF	2.85	River	-	11	270.27
2022	CF	3.54	River	-	11	282.45
2022	CF	3.33	River	0.014	42	1.68
2022	CF	2.97	River	0.014	42	4.96
2022	CF	3.75	River	0.014	42	12.18
2022	CF	6.11	River	0.014	42	13.60
2022	CF	3.15	River	0.014	42	15.07
2022	CF	6.90	River	0.014	42	18.07
2022	CF	3.71	River	0.014	42	50.24
2022	CF	1.99	River	0.014	42	722.70
2022	CF	1.58	River	0.014	42	727.01
2022	CF	1.20	River	0.014	42	731.80
2022	CF	1.41	River	0.014	42	740.57
2022	CF	1.03	River	0.014	42	774.35
2022	CF	1.61	River	0.014	42	778.99
2022	CF	4.03	River	0.056	42	76.77
2022	CF	3.39	River	0.056	42	171.98
2022	CF	1.99	River	0.056	42	384.56
2022	CF	3.65	River	0.058	42	74.83
2022	CF	3.12	River	0.058	42	94.14
2022	CF	2.74	River	0.058	42	107.30
2022	CF	2.90	River	0.058	42	250.57
2022	CF	2.19	River	0.058	42	276.42
2022	CF	2.76	River	0.058	42	291.81
2022	CF	1.55	River	0.057	67	752.79
2022	CF	2.41	River	0.057	67	941.81
2022	CF	1.92	River	0.057	67	951.04
2022	CF	4.72	River	0.051	191	2.57

Year	Prey Type	Se	Water Source	Inflow Velocity (m^3/s)	Marsh Size (ha)	Distance to Inflow (m)
2022	CF	4.03	River	0.051	191	101.36
2022	CF	4.02	River	0.051	191	628.88
2022	CF	1.68	River	0.051	191	653.36
2022	CF	2.36	River	0.056	191	4373.70
2022	CF	1.96	River	0.056	191	4429.42
2022	CF	2.18	River	0.056	191	4441.07
2020	CF	3.49	Ag	0.043	48	440.29
2020	CF	4.49	Ag	0.043	48	440.29
2020	CF	4.50	Ag	0.043	48	440.29
2020	CF	4.05	Ag	0.043	48	614.41
2020	CF	5.14	Ag	0.043	48	614.41
2020	CF	5.31	Ag	0.043	48	614.41
2020	CF	6.56	Ag	0.043	48	614.41
2020	CF	6.72	Ag	0.043	48	614.41
2020	CF	2.84	Ag	0.043	48	857.09
2020	CF	3.32	Ag	0.043	48	857.09
2020	CF	3.89	Ag	0.043	48	857.09
2020	CF	3.44	Ag	0.046	48	496.56
2020	CF	4.57	Ag	0.046	48	496.56
2020	CF	7.05	Ag	0.046	48	496.56
2020	CF	4.64	Ag	0.246	137	322.97
2020	CF	2.81	Ag	0.246	137	363.00
2020	CF	3.00	Ag	0.246	137	363.00
2020	CF	4.54	Ag	-	137	95.47
2020	CF	4.64	Ag	-	137	95.47
2020	CF	5.17	Ag	-	137	95.47
2020	CF	5.65	Ag	-	137	95.47
2020	CF	6.02	Ag	-	137	95.47
2020	CF	6.30	Ag	-	137	143.10
2020	CF	6.63	Ag	-	137	143.10

Year	Prey Type	Se	Water Source	Inflow Velocity (m^3/s)	Marsh Size (ha)	Distance to Inflow (m)
2021	CF	4.11	Ag	0.008	12	22.41
2021	CF	4.02	Ag	0.008	12	40.90
2021	CF	4.26	Ag	0.008	12	54.87
2021	CF	6.04	Ag	0.008	12	66.51
2021	CF	5.61	Ag	0.008	12	80.07
2021	CF	4.20	Ag	0.008	12	80.86
2021	CF	3.54	Ag	0.024	48	872.44
2021	CF	3.25	Ag	0.024	48	882.54
2021	CF	3.38	Ag	0.024	48	890.78
2021	CF	4.66	Ag	0.031	48	678.31
2021	CF	5.00	Ag	0.032	48	567.65
2021	CF	4.63	Ag	0.032	48	644.97
2021	CF	3.88	Ag	0.032	48	668.03
2021	CF	3.71	Ag	0.033	48	1012.32
2021	CF	4.09	Ag	0.033	48	1012.32
2021	CF	3.54	Ag	0.036	48	536.38
2021	CF	4.77	Ag	0.040	48	661.99
2021	CF	5.44	Ag	0.040	48	680.06
2021	CF	3.66	Ag	0.040	48	894.12
2021	CF	7.18	Ag	0.226	137	742.33
2021	CF	2.77	Ag	0.250	137	8.31
2021	CF	3.34	Ag	0.250	137	25.60
2021	CF	2.64	Ag	0.250	137	37.79
2021	CF	3.50	Ag	0.250	137	92.70
2021	CF	6.04	Ag	0.266	137	501.49
2021	CF	3.27	Ag	0.091	856	13.53
2021	CF	5.03	Ag	0.091	856	317.14
2021	CF	2.28	Ag	0.137	856	108.62
2021	CF	2.27	Ag	0.141	856	73.78
2021	CF	10.10	Ag	0.155	856	264.63

Year	Prey Type	Se	Water Source	Inflow Velocity (m^3/s)	Marsh Size (ha)	Distance to Inflow (m)
2021	CF	9.31	Ag	0.155	856	316.50
2021	CF	6.72	Ag	0.155	856	362.82
2021	CF	2.79	Ag	0.238	856	89.24
2021	CF	3.35	Ag	0.261	856	85.65
2021	CF	3.07	Ag	0.261	856	120.24
2022	CF	5.23	Ag	0.012	12	24.50
2022	CF	5.24	Ag	0.012	12	79.41
2022	CF	4.73	Ag	0.012	12	85.45
2022	CF	4.14	Ag	0.025	12	438.51
2022	CF	4.33	Ag	0.068	48	655.36
2022	CF	3.07	Ag	0.068	48	671.43
2022	CF	4.24	Ag	0.070	48	458.50
2022	CF	3.63	Ag	0.070	48	474.69
2022	CF	3.63	Ag	0.070	48	483.05
2022	CF	4.42	Ag	0.194	856	37.30
2022	CF	3.63	Ag	0.194	856	40.87
2022	CF	3.55	Ag	0.224	856	7.76
2022	CF	2.65	Ag	0.224	856	8.71
2022	CF	2.80	Ag	0.224	856	39.25
2022	CF	1.43	Ag	0.260	856	123.01
2022	CF	3.25	Ag	0.260	856	127.28
2022	CF	3.26	Ag	0.260	856	129.64
2022	SM	2.13	Spring	-	66	135.32
2022	SM	6.57	Spring	-	66	209.53
2022	SM	4.17	Spring	-	66	209.59
2022	SM	4.25	Spring	-	66	212.12
2022	SM	4.01	River	-	11	7.85
2022	SM	3.08	River	-	11	7.87
2022	SM	5.50	River	-	11	272.35
2022	SM	5.32	River	-	11	272.69

Year	Prey Type	Se	Water Source	Inflow Velocity (m^3/s)	Marsh Size (ha)	Distance to Inflow (m)
2022	SM	3.79	River	0.014	42	8.13
2022	SM	1.99	River	0.014	42	740.32
2022	SM	5.69	River	0.056	42	76.77
2022	SM	11.60	Ag	0.012	12	24.50
2022	SM	13.90	Ag	0.012	12	79.41
2022	SM	12.40	Ag	0.012	12	85.45
2022	SM	16.60	Ag	0.025	12	438.51
2022	SM	19.40	Ag	0.029	12	1.68
2022	SM	15.50	Ag	0.029	12	4.72
2022	SM	16.80	Ag	0.029	12	87.10
2022	SM	3.97	Ag	0.194	856	40.87
2022	SM	4.50	Ag	0.224	856	8.71
2022	SM	2.05	Ag	0.266	856	931.88
2022	CC	5.35	River	0.006	4	61.63
2022	CC	6.38	River	0.006	4	82.13
2022	CC	6.09	River	0.006	4	95.78
2022	CC	5.37	River	0.029	4	4.30
2022	CC	0.13	River	0.029	4	10.35
2022	CC	4.64	River	0.029	4	11.49
2022	CC	4.18	River	0.029	4	292.05
2022	CC	5.26	River	0.029	4	292.63
2022	CC	5.27	River	-	11	118.83
2022	CC	6.03	River	-	11	272.35
2022	CC	8.80	River	0.014	42	12.18
2022	CC	6.57	River	0.014	42	18.07
2022	BG	3.43	River	-	11	272.69
2022	BG	3.51	River	0.056	67	951.12
2022	BG	3.15	River	0.056	67	957.20
2022	BG	2.21	River	0.057	67	752.79
2022	BG	2.78	River	0.057	67	941.81

Year	Prey Type	Se	Water Source	Inflow Velocity (m^3/s)	Marsh Size (ha)	Distance to Inflow (m)
2022	BG	2.16	River	0.057	67	951.03
2022	MT	13.70	Ag	0.025	12	438.51
2022	MT	7.79	Ag	0.029	12	1.68
2022	MT	9.44	Ag	0.029	12	4.72
2022	MT	10.00	Ag	0.029	12	87.10
2022	OF	5.87	River	0.051	191	2.57
2022	OF	3.92	Ag	0.260	856	127.28
2022	OF	6.11	Ag	0.260	856	127.28
2022	SH	7.30	Spring	-	66	135.32
2022	SH	8.86	Spring	-	66	209.53
2022	SH	7.38	River	0.029	4	4.30
2022	SH	4.61	River	0.029	4	292.05
2022	SH	5.32	River	0.014	42	8.13
2022	SH	10.10	River	0.056	67	957.20
2022	SH	2.98	River	0.057	67	941.81
2022	SH	2.85	River	0.057	67	951.03
2022	SH	4.23	Ag	0.070	48	474.69
2022	SH	10.00	Ag	0.070	48	483.05
2022	SH	9.70	Ag	0.194	856	22.17
2022	SH	10.60	Ag	0.194	856	37.30
2022	SH	11.30	Ag	0.194	856	40.87
2022	SH	7.14	Ag	0.224	856	7.76
2022	SH	7.19	Ag	0.224	856	8.71
2022	SH	9.92	Ag	0.224	856	39.25
2022	SH	4.13	Ag	0.260	856	123.01
2022	SH	5.62	Ag	0.260	856	127.28
2022	SH	5.00	Ag	0.260	856	129.64
2022	SH	6.19	Ag	0.278	856	71.92
2022	SH	4.76	Ag	0.278	856	73.76
2022	SH	4.65	Ag	0.278	856	99.86

Year	Prey Type	Se	Water Source	Inflow Velocity (m^3/s)	Marsh Size (ha)	Distance to Inflow (m)
2022	BT	4.81	Spring	-	66	128.43
2022	BT	13.10	Spring	-	66	209.59
2022	BT	19.80	Spring	-	66	212.12
2022	BT	6.01	River	0.029	4	4.30
2022	BT	6.42	River	0.029	4	10.35
2022	BT	7.47	River	0.029	4	11.49
2022	BT	7.03	River	0.029	4	290.27
2022	BT	5.13	River	0.029	4	292.05
2022	BT	8.42	River	0.029	4	292.63
2022	BT	11.70	River	-	11	272.69
2022	BT	3.47	River	0.014	42	5.92
2022	BT	1.81	River	0.014	42	740.58
2022	BT	3.66	River	0.056	67	951.12
2022	BT	11.60	Ag	0.068	48	655.36
2022	BT	7.22	Ag	0.266	856	914.42
2022	BT	5.83	Ag	0.278	856	99.86
2022	IN	4.90	Spring	-	66	128.43
2022	IN	1.58	Spring	-	66	135.32
2022	IN	2.07	River	-	11	220.17
2022	IN	1.82	River	-	11	247.37
2022	IN	3.51	River	-	11	263.74
2022	IN	4.43	River	0.014	42	722.70
2022	IN	2.34	River	0.014	42	724.98
2022	IN	1.92	River	0.014	42	727.01
2022	IN	5.37	River	0.014	42	731.80
2022	IN	11.80	Ag	0.012	12	79.41
2022	IN	4.80	Ag	0.025	12	474.95
2022	IN	3.54	Ag	0.068	48	671.43
2022	IN	1.62	Ag	0.263	856	1005.23
2022	IN	1.22	Ag	0.266	856	914.42

Year	Prey Type	Se	Water Source	Inflow Velocity (m^3/s)	Marsh Size (ha)	Distance to Inflow (m)
2022	IN	4.81	Ag	0.278	856	71.92
2022	IN	2.84	Ag	0.278	856	99.86

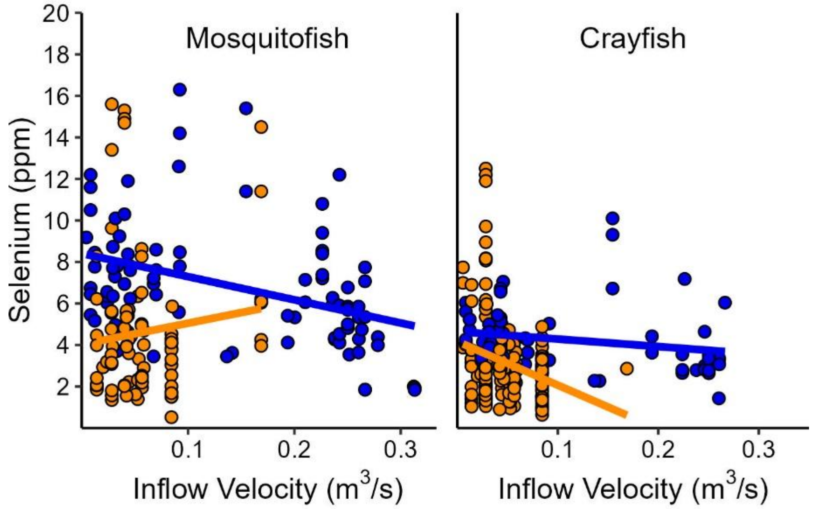

Fig. 1 Relationship of mosquitofish and crayfish selenium concentrations (ppm dw) to the inflow velocity (two-week rolling average at prey capture date) in river-fed (orange) and ag-fed (blue) marshes at the Salton Sea, California, USA (2020–2022)

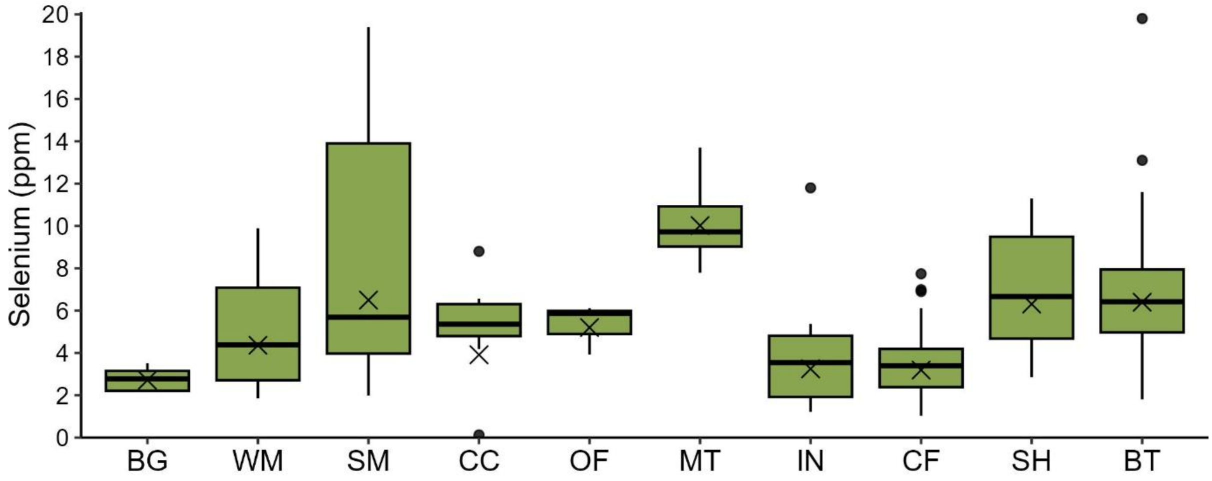

Fig. 2 Comparison of selenium concentrations (ppm dw) in prey sampled from spring-fed, river-fed, and ag-fed marshes (combined) at the Salton Sea, California, USA (2022). BG = bluegill sunfish (*Lepomis macrochirus*); WM = western mosquitofish (*Gambusia affinis*); SM = sailfin molly (*Poecilia latipinna*); CC = common carp (*Cyprinus carpio*); OF = other fish (*Cyprinella*, *Micropterus*, *Morone* spp); MT = Mozambique tilapia (*Oreochromis mossambicus*); IN = Belostomatidae, Corixidae spp, and *Coleoptera* sp; CF = red swamp crayfish (*Procambarus clarkii*); SH = shrimp (*Palaemonidae* sp); BT = American bullfrog tadpoles (*Lithobates catesbeianus*). Boxplots illustrate the 25th and 75th percentiles, with medians represented by lines inside each box. Whiskers extend to the 5th and 95th percentiles. Dots denote outliers and X's indicate geometric means
